# Diet and environmental factors jointly drive the gut microbiome, resistome, and virulome of urban bats

**DOI:** 10.1038/s41522-026-00930-y

**Published:** 2026-02-04

**Authors:** Long Huang, Ying-Ting Pu, Yan-Hui Zhao, Xiao-Yu Sun, Yue Zhu, Ya-Ping Lu, Hai-Xia Leng, Jiang Feng, Long-Ru Jin, Ke-Ping Sun

**Affiliations:** 1 Jilin Provincial Key Laboratory of Animal Resource Conservation and Utilization, Northeast Normal University, Changchun, China; 2 Key Laboratory of Vegetation Ecology, Ministry of Education, Changchun, China; 3 Jilin Provincial International Cooperation Key Laboratory for Biological Control of Agricultural Pests, Changchun, China; 4 Jilin Engineering Laboratory for Avian Ecology and Conservation Genetics, School of Life Sciences, Northeast Normal University, Changchun, China

**Keywords:** Ecology, Ecology, Microbiology

## Abstract

The coexistence and horizontal transfer of antibiotic resistance genes (ARGs) and virulence factor genes (VFGs) carried by urban wildlife represent an emerging form of biological pollution, constituting a significant threat to public health. We employed meta-omic approaches to evaluate the effects of host traits (sex, age, etc.), environmental factors (including geographical location and time), and diet (including food composition and antibiotic residues) on the bacterial, ARG, and VFG profiles of *Vespertilio sinensis*, an urban-dwelling bat. Our results demonstrate that the feces of *V. sinensis* harbor diverse ARGs and VFGs, but their genomic evidence for horizontal mobility in bacterial communities is limited. Notably, environmental changes over time and across geographical locations are associated with the ARG and VFG profiles, potentially due to the influence of pollutants in specific habitats. Dietary factors are associated with their dynamics through the microbiome, with antibiotic residues exerting selective pressure on ARG profiles. No significant impacts of sex, age, body size, and reproductive status on the gut microbiota, resistome, or virulome were observed. This study provides valuable insights into the ecological drivers of the gut microbiome, resistome, and virulome in bats, thereby contributing to our understanding of the public health risks associated with urban wildlife.

## Introduction

In the post-antibiotic era, the regional and global dissemination of antibiotic resistance posed a significant threat to public health security. While extensive research has focused on the spread and impact of antibiotic resistance in clinical and agricultural settings, the role of wildlife as reservoirs and vectors of antibiotic resistance genes (ARGs) and antibiotic-resistant bacteria (ARB) remain underexplored. In the context of ‘One Health’, the health of humans, wildlife, and ecosystems is interconnected. The gut of wild animals serves as a reservoir for pathogenic microorganisms and resistance genes^1^. Under the high selection pressure exerted by human antibiotics use, antibiotics and antimicrobial resistance spill over into wildlife in underdeveloped or even primitive environments^2–4^. Environmental pollutants, including antibiotics and ARGs, are ingested by animals and accumulated at higher trophic levels through nutrient transfer^5^, subsequently inducing microbial resistance via horizontal gene transfer (HGT) facilitated by mobile genetic elements (MGEs) or selection pressure. The increased use of organic manure, environmental pressures, and human-wildlife interactions during urbanization often promote this process^6–9^. Virulence factor genes (VFGs) contribute to bacterial pathogenicity, and their coexistence and HGT with ARGs exacerbate the risk of biological contamination^10–12^. With the increasing interactions between humans and wildlife, it is crucial to investigate the distribution and coexistence of ARGs and VFGs within the gut microbiota of wildlife, as well as the factors influencing these processes.

Given that the gut microbiota hosts ARGs and VFGs, various factors influencing the gut microbiome of wildlife—such as host traits (including genetics^13^, age^14^, sex^15^, reproductive status^16^, and social interactions^17^), diet^18–20^, and environmental conditions (e.g., season^21–23^ and urbanization^7^)—are expected to indirectly affect the resistome. Among these, diet-related antibiotic exposure may exert the most significant influence by directly selecting ARB and driving the evolution of antibiotic resistance^24,25^. Moreover, phylogenetic boundaries and fitness costs limit the cross-community migration of ARGs and VFGs^26,27^, resulting in uneven distribution and host preferences. However, the mobility facilitated by MGEs across diverse species complicates the association of ARGs and VFGs with specific hosts^28^. The combined and cumulative effects of these factors heighten the unpredictability of ARGs and VFGs distribution patterns. Consequently, establishing clear associations between various confounding factors, gut microbes, resistome, and virulome presents a substantial challenge, particularly in non-laboratory settings.

Urban land expansion leads to the loss and fragmentation of wildlife habitats, exacerbating resource competition, disease transmission, and property damage between humans and wildlife. Following the COVID-19 pandemic, there has been a growing focus on wildlife inhabiting urban environments, particularly nocturnal and elusive species such as bats^7,18,29^. The feeding ecology of insectivorous bats—characterized by high foraging efficiency, substantial food intake, and expansive home ranges—results in extensive environmental interactions and microbial exchange, positioning them as key indicators of environmental pollution^30,31^. *Vespertilio sinensis* demonstrates a preference for cohabitation with humans, particularly during the summer, when they form maternal communities and exhibit cooperative behavior in rearing offspring. This pattern of community aggregation and cooperative reproduction fosters social interactions and microbial exchange^32^, potentially increasing exposure to a broader reservoir of antibiotic resistance and pathogenic agents. Furthermore, the substantial physiological changes and complex environmental stressors encountered before and after parturition challenge the stability of the gut microbiota^16,33,34^, as well as to the health of both host and offspring^34,35^. These factors collectively drive temporal changes in the gut microenvironment, influencing gut microecology in a personalized manner^36,37^. However, considerable gaps in knowledge regarding the characteristics, time dynamics, and drivers of gut microbiota and the resistome during pregnancy and lactation hinder our understanding of reproductive health and hygiene risks in wildlife.

In this study, we characterized the temporal dynamics of gut microbiota, resistome, and virulome within the maternal community of *V. sinensis* inhabiting urban areas during pregnancy and lactation, employing longitudinal metagenomic sequencing. We considered multiple factors, including age, sex, reproductive status, time, and geographical location, and incorporated subadults and rural populations for comparative analysis. Using DNA metabarcoding, we assessed dietary variations among these bats and quantified antibiotic residues in fecal samples to evaluate the impact of diet-related antibiotic stress. Our objective was to evaluate variations in the microbiome, resistome, and virulome, and to elucidate how specific factors contribute to shifts in antibiotic resistance and pathogenicity within the context of the One Health framework. We hypothesize that environmental and dietary factors, rather than host traits, primarily shape the microbiome, resistome and virulome.

## Results

### Metagenomic analysis reveals comprehensive profiles of ARGs, VFGs, and bacterial communities

**Fig. 1:**
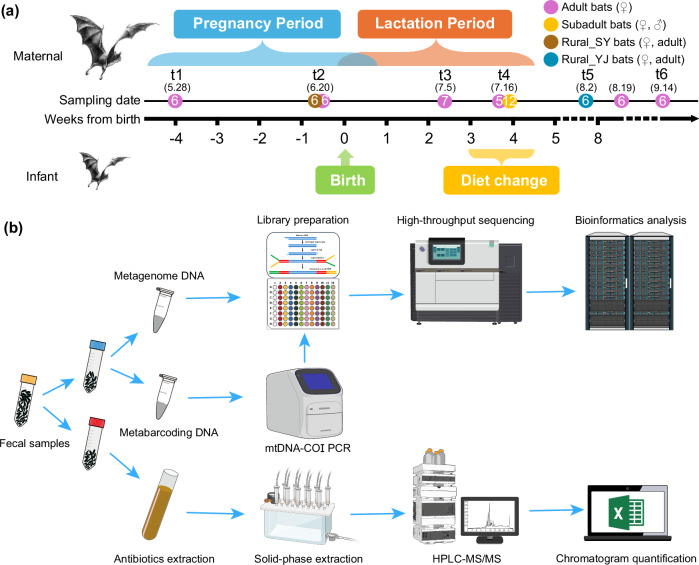
Overview of sampling design and experimental procedures. **a** Sampling settings covering the period of pregnancy, littering, lactation, and weaning in bats. The breeding period of bat populations was defined through field observations. Numbers in parentheses indicate sampling dates, while numbers within circle represent sample sizes. **b** A concise workflow illustrating the conversion of fecal samples into biological data.

**Fig. 2:**
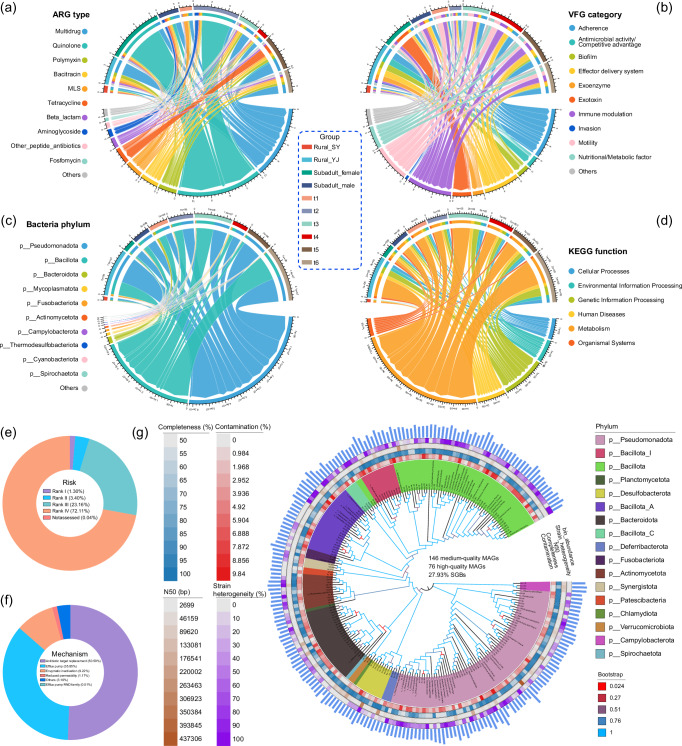
A panoramic view of gut bacteria, resistome, and virulome in *Vespertilio sinensis.* The chordal graph illustrates the composition of ARG types (**a**), VFG categories (**b**), bacterial phyla (**c**), and KEGG functions (**d**) across various groups. The donut chart shows the proportions of ARG risks (**e**) and resistance mechanisms (**f**) across all samples. **g** Phylogenetic tree of 222 bacterial MAGs. Circular heatmaps on the outer ring of the tree display contamination, completeness, N50, strain heterogeneity, from inner to outer layers. The outermost blue bars represent the log-transformed abundance of each MAG.

A total of 60 fecal samples, which included metagenomic data, antibiotic concentrations, and eDNA metabarcoding, were analyzed from male subadult, female subadult, and female adult bats (Fig. 1). The analysis of 4.4 billion reads facilitated the detailed identification of ARG characteristics across all samples, encompassing 27 types and 1179 subtypes. The predominant ARGs conferred resistance to multidrug (29.74%), quinolone (29.10%), polymyxin (7.89%), bacitracin (7.35%), macrolide-lincosamide-streptogramin (MLS) (6.47%), and tetracycline (6.07%) (Fig. 2a). The total abundance of ARGs in each sample ranged from 0.15 to 21.71 copies/cell, with an average of 4.53 copies/cell. Overall, low-risk ARGs were the most prevalent, comprising 72.11% in rank Ⅳ and 23.16% in rank Ⅲ (Fig. 2e). The primary resistance mechanisms included antibiotic target replacement (50.59%), efflux pump (35.85%), and enzymatic inactivation (9.22%) (Fig. 2f). In terms of VFGs, all categories in the database were detected within the samples, with dominant categories including immune modulation (19.10%), adherence (19.09%), motility (16.68%), effector delivery system (14.27%), nutritional/metabolic factor (10.23%), and exotoxin (7.06%) (Fig. 2b). The total abundance of VFGs ranged from 0.09 to 22.87 copies/cell, with a mean of 5.45 copies/cell. Across all samples, bacterial sequences accounted for an average of 42.35% of the clean reads after annotation. The gut microbiota of *V. sinensis* consisted of 49 bacterial phyla, with Pseudomonadota, Bacillota, Bacteroidota, and Mycoplasmatota representing the top four taxonomic groups, accounting for 98.33% of the total bacterial community (Fig. 2c). The most abundant genera included *Clostridium* (17.13%), *Klebsiella* (11.75%), *Enterobacter* (11.57%), *Lactococcus* (10.04%), and *Escherichia* (5.92%) (Supplementary Fig. 1).

The co-assembly of clean reads from all samples resulted in 6,091,355 contigs, encompassing 8,970,773 genes for further analysis. Following clustering at 95% nucleotide sequence identity, a non-redundant microbial gene catalog was generated, containing 7,758,182 genes with an average length of 459 bp. Functional annotation using KEGG revealed that the most abundant categories at level 1 were ‘metabolism’ and ‘genetic information processing’ (Fig. 2d). At level 2, ortholog groups primarily associated with carbohydrate metabolism, amino acid metabolism, energy metabolism, nucleotide metabolism, membrane transport, metabolism of cofactors and vitamins, transcription, information processing in viruses, and lipid metabolism were identified (Supplementary Fig. 2).

The metagenomic binning resulted in the identification of 702 bacterial bins, including 146 of medium-quality and 76 of high-quality metagenome-assembled genomes (MAGs) (Fig. 2g). The distribution of dominant bacteria in the gut of *V. sinensis* was determined based on the taxonomic classification of MAGs with medium to high quality. The summary statistical data of bacterial MAGs has been provided in Supplementary Table 1. The most abundant phylum was Pseudomonadota, followed by Bacillota. Additionally, Bacteroidota, Bacillota_A, Actinomycetota, Bacillota_I, and Desulfobacterota were also prominent in the sampled microbiome. Approximately 89.19% (198/222) of bacterial MAGs were annotated at the genus level. A considerable portion of the MAGs (62/222, 27.93%) was further classified into species-level genome bins (SGBs).

### Contig assembly and binning decipher the mobility of and bacterial hosts of ARGs and VFGs

**Fig. 3:**
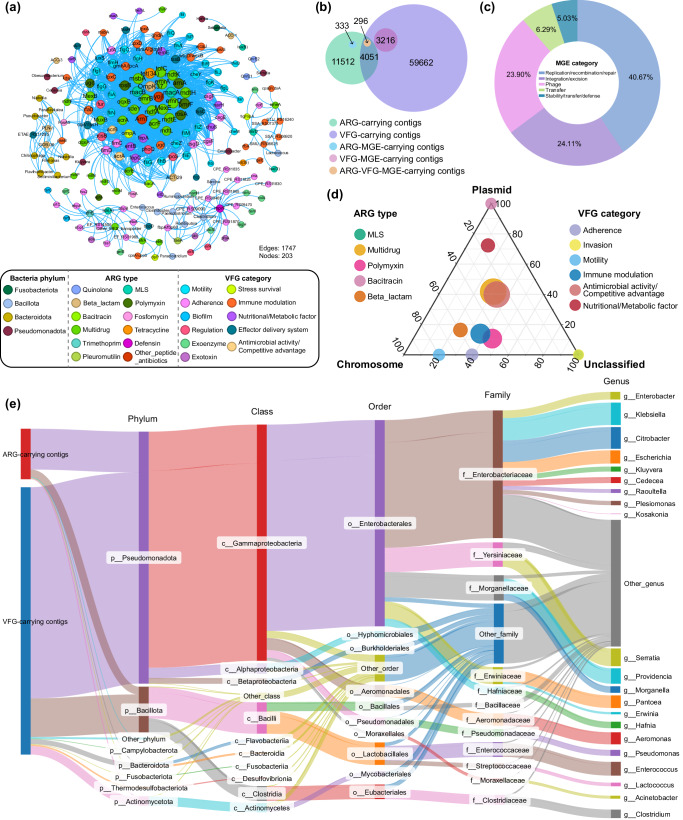
Mobility potential and bacterial hosts of ARGs and VFGs in *Vespertilio sinensis.* **a** Co-occurrence network of bacterial genera, ARG subtypes, and VFG genes. Nodes are colored by type, with node size proportional to the number of connections. The network was constructed using connections with a Spearman correlation coefficient greater than 0.8 and a *p*-value less than 0.01 to ensure statistical significance, displaying only positive correlations. **b** The Venn diagram depicts the intersections among ARG/VFG/MGE-carrying contigs. The size of each circle is proportional to the number of contigs. **c** The donut chart illustrates the composition of MGE categories on ARG-VFG-carrying contigs. **d** The ternary plot shows the proportion of ARGs and VFGs distributed across plasmids and chromosomes. **e** The Sankey diagram illustrates the taxonomy of the dominant microbial genera associated with ARGs and VFGs. The height of each rectangle represents the number of the ARG/VFG-carrying contigs.

The co-occurrence network revealed intricate, abundance-based correlations between ARGs, VFGs, and microbial genera (Fig. 3a). ARGs conferring resistance to multidrug, polymyxin, and MLS antibiotics were found to form a tighter interconnected module with VFG categories related to motility, adherence, immune modulation, and nutritional/metabolic factors. Twenty-four bacterial genera from Bacillota, Bacteroidota, Pseudomonadota, and Fusobacteriota were identified as potential hosts of both ARGs and VFGs, with *Clostridium* showing the most frequent association. Moreover, specific ARG subtypes, including *tet(34)*, *OmpK37*, *macA*, *macB*, *mdtK*, *mdtH*, *emrA*, *mprF*, *emeA*, and *dfrE*, were found to coexist with various VFGs. Notably, two VFGs (*ETAE_RS 11285* and *pseB*) and two ARGs (*GOB-36* and *PLN-1*) exhibited the broadest range of bacterial hosts.

The assembly and annotation of the flanking regions of ARGs and VFGs elucidated the genetic relationships between target genes and enhanced the accuracy of host identification. Among all ARG-carrying contigs, 35.19% were found to carry VFGs, while 2.57% harbored both VFGs and MGEs (Fig. 3b). MGEs physically linked to both ARG and VFG were classified into five categories: replication/recombination/repair (40.67%), integration/excision (24.11%), phage (23.90%), transfer (6.29%), and stability/transfer/defense (5.03%) (Fig. 3c). Genetic location analysis revealed that the majority of ARG types and VFG categories are situated on chromosomes (Fig. 3d), with ARG-VFG-carrying plasmids classified as either nonmobilizable (99.42%) or mobilizable (0.58%), with a notable absence of conjugative plasmids. The output of MOBFinder showed that the accuracy of plasmid classification (mob_score > 0.5) reaches 98.15%.

To further investigate the microbial sources of ARGs and VFGs observed in the bat gut, taxonomic assignment was conducted on ARG- and VFG-carrying contigs. The co-occurrence of ARGs and VFGs was detected in a variety of microorganisms, identified as pathogenic antibiotic-resistant bacteria (PARBs; carrying both ARGs and VFGs). This group included *Citrobacter*, *Klebsiella*, *Serratia*, *Providencia*, *Aeromonas*, *Enterococcus*, among others, suggesting that these pathogenic bacteria may possess resistance to common antibiotics (Fig. 3e). MGE-carrying PARBs, which involve four genera of Bacillota and 32 genera of Pseudomonadota, underscored the potential risk of horizontal transfer of both antibiotic resistance and virulence (Supplementary Fig. 3).

### Age and sex have a minimal impact on diversity of ARG, VFG, and bacterial communities

To assess the effects of age on ARG, VFG, and bacteria diversity in the gut, we compared samples from female subadults (group: Subadult_female) and from female adults (group: t4) collected concurrently. Surprisingly, both the total abundance and diversity of ARG and VFG in subadult bats were comparable to those in adult bats (Supplementary Fig. 4). NMDS analysis based on Bray-Curtis distance revealed no distinct separation of ARG, VFG, and bacterial communities by age (ARG: Stress = 0.077, *R*^2^ = 0.155, *p* = 0.146; VFG: Stress = 0.022, *R*^2^ = 0.082, *p* = 0.736; Bacteria community: Stress = 0.137, *R*^2^ = 0.127, *p* = 0.072; KEGG function: Stress = 0.039, *R*^2^ = 0.155, *p* = 0.063; Supplementary Figs. 4 and 5). Similarly, a comparison between subadult females and males revealed no sex-related differences (Supplementary Figs. 6 and 7). In conclusion, age and sex exert minimal influence on the diversity and community structure of ARG, VFG, and bacteria.

### Geographic location explains changes in ARGs, VFGs, and bacterial communities in the gut

**Fig. 4:**
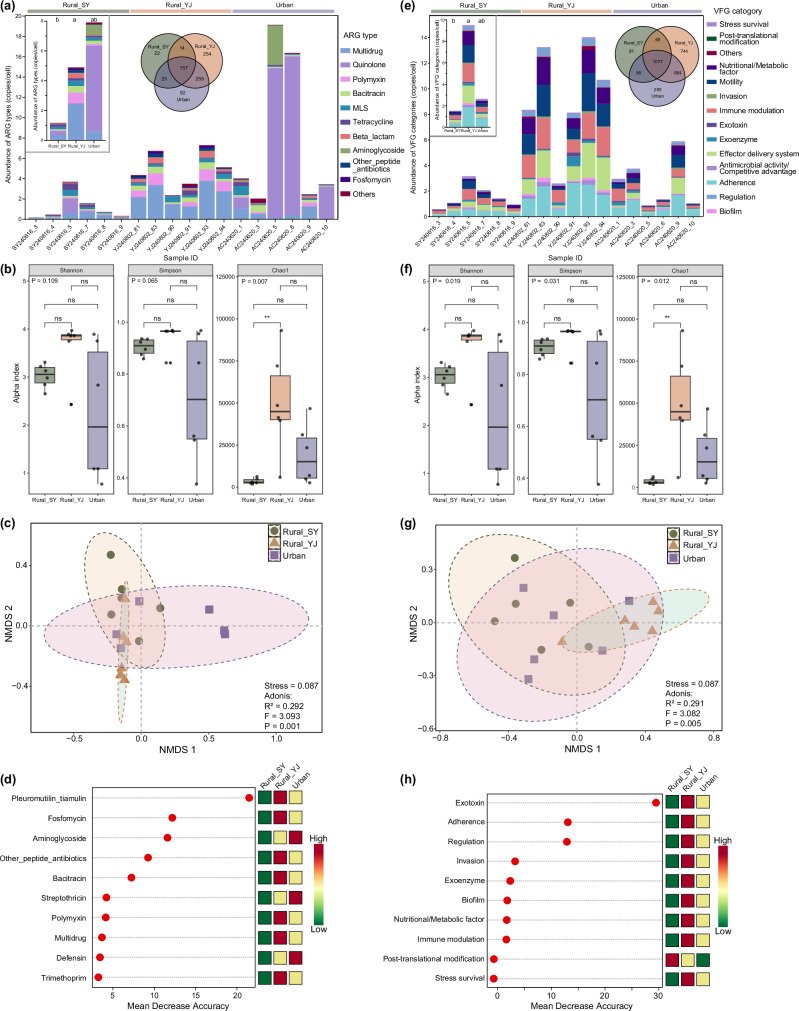
Variation of ARG and VFG profiles in samples from Rural_SY (*n* = 6), Rural_YJ (*n* = 6), and Urban (*n* = 6). The stacked bar chart displays the dominant composition of ARGs (**a**) and VFGs (**e**) in each sample from three locations. In the subgraph, the stacked bar chart shows the differences in mean values among the three groups, while the Venn diagram illustrates the number of shared and unique ARGs and VFGs across these groups. The box plot displays the differences in alpha diversity of ARGs (**b**) and VFGs (**f**) among the three groups. Significance level: ns, not significant; *, *p* < 0.05; **, *p* < 0.01. Non-metric multidimensional scaling (NMDS) ordinations reveal the differences in the community structure of ARGs (**c**) and VFGs (**g**). Random forest analysis identifies ARG types (**d**) and VFG categories (**h**) that significantly contribute to community differences. Distinct color blocks represent the average abundance of each feature.

The comparison between urban sites (group: t2) and two rural sites (group: Rural_SY and Rural_YJ) revealed significant differences in the abundance and diversity of ARGs, VFGs, and bacterial communities (Fig. 4a and Supplementary Fig. 8). Specifically, Rural_SY exhibited lower levels of both ARGs and VFGs compared to urban samples (Fig. 4a, e). In contrast, Rural_YJ demonstrated the most diverse and abundant assemblage of ARGs and VFGs. Random forest analysis identified key ARG types, such as pleuromutilin_tiamulin, notable VFG categories, like exotoxin, and bacterial genera, such as *Liberibacter*, across different locations (Fig. 4d, h and Supplementary Fig. 8). Regarding bacterial composition, Rural_SY had the fewest bacterial species but showed more even distribution than Rural_YJ and urban samples (Supplementary Fig. 8b). Meanwhile, Rural_YJ and urban samples harbored numerous rare bacteria and exhibited greater functional diversity (Supplementary Figs. 8a, b and 9). In summary, the observed differences among the three locations primarily reflect the influence of geographic location on bacterial composition, resistome, and virulome, rather than the influence of urban-rural divide.

### Longitudinal variations in abundance and diversity reflect the association between ARG, VFG, and bacterial communities

**Fig. 5:**
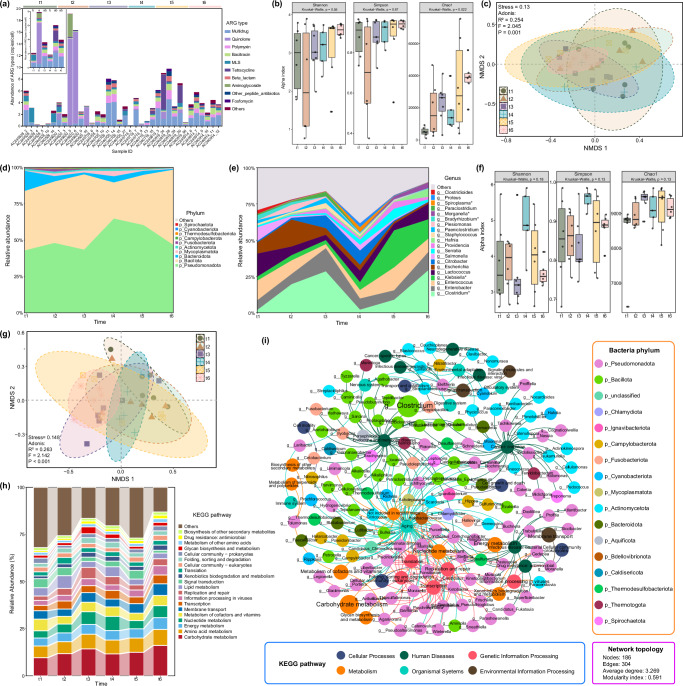
Longitudinal changes in ARG and bacterial profiles in *Vespertilio sinensis* over time. The stacked bar chart and area chart illustrate the composition of dominant ARG types (**a**), bacterial phyla (**d**), and bacterial genera (**e**). The box plot shows differences in alpha diversity of ARGs (**b**) and bacteria (**f**) among six time points. Non-metric multidimensional scaling (NMDS) ordinations reveal the differences in the community structure of ARGs (**c**) and bacteria (**g**). **h** Changes in relative abundance of KEGG functions among six time points. **i** Co-occurrence network of bacterial genera and KEGG functions. Nodes are colored by type, with node size proportional to the relative abundance. The network was constructed using connections with a Spearman correlation coefficient greater than 0.8 and a *p*-value less than 0.01 to ensure statistical significance, displaying only positive correlations.

To explore temporal patterns associated with reproductive status, samples collected at six time points (t1: *n* = 6; t2: *n* = 6; t3: *n* = 7; t4: *n* = 5; t5: *n* = 6; t6: *n* = 6) were compared. While the total abundance and diversity of ARGs and VFGs exhibited a general increasing trend over time, alpha diversity unexpectedly decreased during the t4 stage (Fig. 5a–c and Supplementary Fig. 10). NMDS analysis revealed that the community structures of both ARGs and VFGs responded to temporal variations from t1 to t6 (ARG: Stress = 0.130, *R*^2^ = 0.254, *p* = 0.001; VFG: Stress = 0.108, *R*^2^ = 0.244, *p* = 0.005; Fig. 5c and Supplementary Fig. 10c). The similar trends observed in ARGs and VFGs suggest they may be influenced by common factors, such as changes in the host microbiota.

The investigation into the bacterial community revealed significant changes in community structure from t1 to t6 (Stress = 0.148, *R*^2^ = 0.263, *p* < 0.001; Fig. 5g–j and Supplementary Fig. 11). The abundance of Bacillota, particularly the most abundant genus *Clostridium*, showed notable fluctuations (Fig. 5d, e–g, Supplementary Fig. 12), with the trend closely mirrored the diversity pattern of ARGs and VFGs (Supplementary Fig. 13b). From t3 to t4, bacterial diversity increased while the relative abundance of *Clostridium* decreased (Fig. 5e, f). These results suggested a strong association between the genus *Clostridium* and both ARGs and VFGs. Additionally, to explore the temporal dynamics of microbial function from pregnancy to delivery to lactation, potential patterns of the KEGG pathway were analyzed (Fig. 5h). Functional pathways were grouped into three clusters based on the abundance changes from t1 to t6 (Supplementary Fig. 13a), with pathways related to drug resistance—such as ‘Cationic antimicrobial peptide (CAMP) resistance’, ‘beta-Lactam resistance’, and ‘Vancomycin resistance’—being significantly enriched in clusters 1 and 3 (Supplementary Fig. 14). The abundance patterns of the KEGG level 2 function ‘Drug resistance: antimicrobial’ corresponding to these functions aligned with the trends in the abundance of *Clostridium* and the diversity of ARGs and VFGs (Supplementary Fig. 13b). Subsequently, the association between bacteria, ARGs, and VFGs was further validated through multivariate correlation analysis (Supplementary Fig. 13c). However, the species-function network analysis revealed a significant correlation between the ‘Drug resistance: antimicrobial’ function and the genera *Moranella*, *Superficieibacter*, *Cedecea* and *Candidatus_Fukatsuia*, but not with *Clostridium* (Fig. 5i).

### Diet-mediated antibiotic intake alters resistome through selective effects

**Fig. 6:**
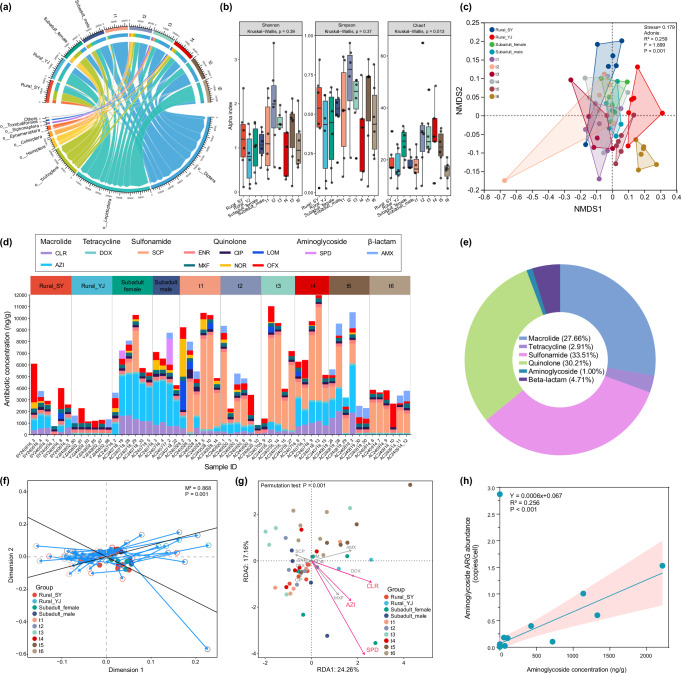
The relationship between dietary composition and antibiotic residues in *Vespertilio sinensis.* **a** The chordal graph shows the composition of insect orders in the diet of bats across different groups. **b** The box plot displays the differences in alpha diversity of dietary composition among different groups. **c** Non-metric multidimensional scaling (NMDS) ordinations reveal the differences in dietary composition among groups. **d** The composition of antibiotics detected in each fecal sample. **e** The dominant antibiotic categories detected in all samples. **f** Procrustes analysis of antibiotics and dietary composition across all groups (M² = 0.868, *p* = 0.001). **g** Redundancy analysis (RDA) shows the impact of antibiotics on ARG composition. **h** Linear correlation between concentration of aminoglycoside antibiotics and the abundance of aminoglycoside ARGs.

To investigate the impact of diet-related factors on the gut resistome, we examined the relationship between dietary composition, antibiotic residues, and ARGs. *V. sinensis* consumes insects from 16 orders, with Diptera (42%), Lepidoptera (34%), Trichoptera (13%), and Hemiptera (4%) being the dominant taxa (Fig. 6a). The dietary composition varied significantly among different groups, with an increase in prey species from pregnancy (t1) to lactation (t4) (Fig. 6b, c). In terms of antibiotics, 12 types from six categories were detected in bat feces, with sulfonamide (33.51%), quinolone (30.21%), and macrolide (27.66%) as the predominant categories (Fig. 6d, e). Antibiotic concentrations in samples from two rural sites were lower than urban samples (Fig. 6d). Surprisingly, the antibiotic concentration in the gut of subadult bats was comparable to those in adult bats. Procrustes analysis revealed a strong correlation between diet composition and antibiotic concentration in the gut (Fig. 6f). The concentrations of spectinomycin dihydrochloride (SPD), clarithromycin (CLR), and azithromycin (AZI) were found to significantly correlated with the ARG composition (*p* < 0.001; Fig. 6g). Additionally, a significant positive correlation was observed between the total concentration of aminoglycosides in bat feces and the total abundance of aminoglycoside resistance genes (*R*^*2*^ = 0.265, *p* < 0.001; Fig. 6h). These findings represent consistency between metagenomics, DNA barcoding, and antibiotic data.

### Gut bacteria, ARGs, and VFGs are collectively related to dietary and environmental factors

**Fig. 7:**
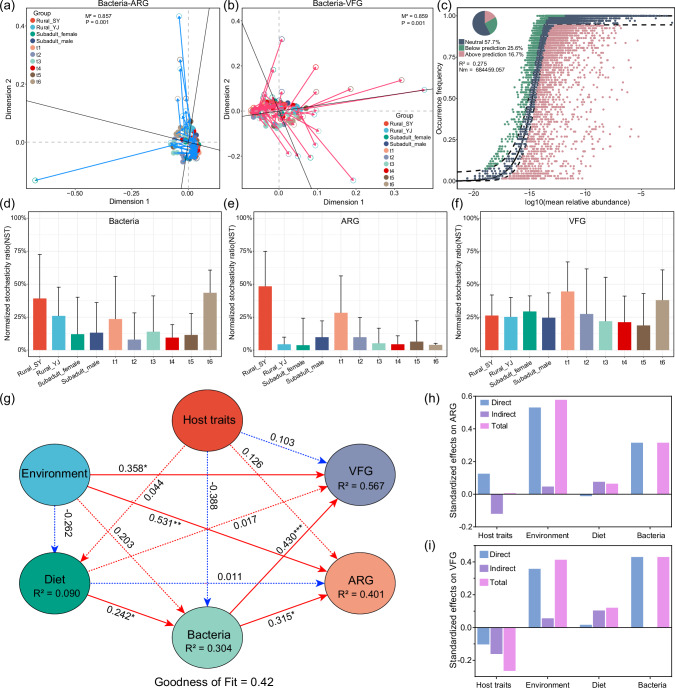
The driving mechanism behind variations in gut bacteria, resistome, and virulome in *Vespertilio sinensis.* Procrustes analysis of the compositions of bacterial genera, ARG subtypes (**a**), and VFG genes (**b**). **c** Fit of the neutral community model (NCM) for bacterial community assembly. The bar chart displays the measurement of the community assembly process for bacteria (**d**), ARG (**e**), and VFG (**f**) using normalized random ratio (NST) index. **g** Partial least squares path modeling (PLS-PM) showing the effects of host traits (age, sex, body size, and reproductive status), environment factors (time and location), and diet (dietary composition and antibiotic residues) on the abundance of gut bacteria, ARGs, and VFGs. Solid lines indicate significant (*p* < 0.05) effects. Significance: *p < 0.05, **p < 0.01, ***p < 0.001. Red lines represent positive effects, and blue lines represent negative effects. The bar chart shows the standardization effects of specific factors on ARG (**h**) and VFG (**i**) profiles.

Procrustes analysis revealed a significant relationship between the composition of microbial genera and the distribution of ARGs and VFGs (*p* = 0.001; Fig. 7a, b). Given the substantial variations in gut microbiota and resistome across different groups, the mechanisms of community assembly were further examined. The results showed that the bacterial community had a relatively low goodness of fit (R^2^ = 0.275) to the neutral model, and the observed frequencies of ARG and VFG deviated from the neutral model (Fig. 7c; Supplementary Fig. 15). Moreover, the null model-based NST values of the three were less than 0.5, suggesting that the changes in ARGs, VFGs, and bacterial communities were primarily driven by deterministic processes (Fig. 7d–f). Further exploration into the driving mechanisms revealed that host characteristics, such as sex and age, do not significantly affect diet, bacterial composition, ARGs, or VFGs (Fig. 7g). In contrast, environmental factors, like geographical location and time, have a direct positive correlation with the abundance and diversity of ARGs and VFGs (Fig. 7g–i). Host traits, environment, diet, and bacteria contribute 40.1% of ARG changes and 56.7% of VFG changes, respectively. The total effects of environmental factors and bacteria are the highest, followed by diet. Furthermore, dietary factors, involving diet diversity and antibiotic residues, are not directly associated with ARGs and VFGs but instead indirectly promote positive effects through linking the diversity of gut bacteria (Fig. 7g). The comprehensive overview of the outer model and inner model can be found in Supplementary Fig. 16 and Table 2, respectively.

## Discussion

Through targeted sampling and metaomics analysis of wild bats living near humans, we systematically examined the association and causal relationships between host traits, environmental factors, diet, bacteria, ARGs, and VFGs. Our findings highlighted the substantial impact of diet and environmental changes on the bacterial composition and the distribution of ARGs and VFGs in the bat gut. In contrast, the influence of host traits (sex, age, body size, and reproductive status) was not observed in this study. Meanwhile, the low horizontal transfer potential of ARGs and VFGs carried by bats suggests limited pressure on the surrounding environment and human health.

Abundant ARGs and VFGs have been identified in the gut of *V*. *sinensis*, with both their total abundance and diversity surpassing those of insectivorous bats dwelling in the wild, as highlighted in our previous study^18^. These levels are comparable to those in antibiotic-contaminated environments such as wastewater, soil, farm workers, and livestock manure^38–42^. The gut resistome of bat is notedly characterized by a higher proportion of multidrug (29.74%) ARGs compared to other flying animals (< 20%)^18,43–45^, and is strongly associated with various VFG categories, suggesting an elevated risk. However, the genetic positioning of ARGs and VFGs on chromosomes or non-mobilizable plasmids, along with their low co-occurrence rate with MGEs, points to a limited potential for horizontal transfer. Similar gene mobility has also been observed in other bat species and wild rodents^11,18^, but the number of PARBs in this study is even fewer. This indicates that even though bat defecation may pose certain hygiene concerns, the resistance and virulence of fecal bacteria are likely to remain confined to a local range. It should be noted that seasonal patterns, climate change^46^, pollutants (such as heavy metals and microplastics)^47^, genetic compatibility, and ecological connectivity^48^ may promote the conjugate transfer of ARGs and VFGs between different environmental compartments.

Wildlife in proximity to human pollution sources is generally more vulnerable to drug resistance and pathogenic bacteria^7,49^. This heightened susceptibility is largely attributed to urbanization, which facilitates complex animal-human-environment interactions and introduces a range of pollutants, including heavy metals, pesticides, drugs, and biological contaminants such as ARGs and VFGs, into the ecosystem. Previous studies have demonstrated that land use changes resulting from urbanization increase pollutant pressure and drive the rise of antibiotic resistance and potential human bacterial pathogens in soil animals^7,50^. In this study, the assembly of bacterial communities, resistome, and virulome driven by deterministic processes suggests the predominance of specific environmental factors, such as the influence of pollutants, over incidental events and random processes. That is to say, wildlife exposed to anthropogenic pollution and adapted to urban ecology are expected to harbor more clinical ARGs and human-related pathogens compared to rural-dwelling animals. Contrary to expectations, however, our analysis revealed an unexpectedly high abundance of ARGs and VFGs in samples from a rural site (Rural_YJ) compared to urban areas. This observation may be influenced by several factors, including deviations in sampling time and location. Especially, rural bats were sampled at two different times (t2 and t6) at separate locations, which could introduce variability due to seasonal patterns, climate factors or site-specific conditions that alter the gut resistome of animals^22,51^. Additionally, another potential explanation for this finding is that pollution levels at the rural sampling site may exceed those at the urban location, potentially having a stronger impact on local bat populations and masking the effects of urbanization due to regional variability. Despite the generally low antibiotic residue levels in rural bats, this observation, suggests the possibility of co-selection driven by pollutants other than antibiotics, such as metals, biocides, and herbicides^52^. Common agricultural pesticides, including heavy metals and fungicides, can increase ARGs, MGEs, and VFGs in the soil-plant-insect food chain and in insectivores through shared resistance mechanisms such as efflux pumps^53,54^. Therefore, a more comprehensive investigation into the relationship between environmental pollution in bat foraging areas and the resistome and virulome is warranted.

Beyond the selection effect and horizontal transfer of environmental pollutants, the gut bacterial composition plays a pivotal role in shaping the resistome and virulome, as reported in previous studies^55,56^. Accordingly, host traits, such as phylogeny, sex, age, and body size, may indirectly correlated with the distribution of ARGs and VFGs in the gut via modifications in microbiota composition^20,57,58^. Sex differences in gut microbial composition, as well as a shared age-related decrease in sex-dependent differences, have been observed in human cohort studies^59^. However, in this study, host traits, including sex, age, and reproductive status did not account for significant variations in the gut microbiome, resistome, or virulome. Despite the potential influence of sex hormones on the gut microbiome, leading to sex-based differences^60,61^, such effects are sometimes confounded or masked by other variables in certain studies^20,62,63^. The gut microbiome and resistome in juveniles are largely shaped by early mother-to-child transmission, with subsequent evolution through age^64,65^. Additionally, dietary and metabolic changes can drive age-dependent assembly of the gut resistome and virulome through alterations in microbial taxonomy^40,65,66^. In this study, subadult bats transitioning from a milk-based diet to an insect-based diet may exhibit a stabilizing microbiota^67^, resulting in ARG and VFG levels comparable to those observed in adults. It is also important to note that the study’s limitations, such as the relatively small sample size and the exclusion of juvenile and adult male bats, may have obscured age- and sex-related effects, which could have acted as potential confounding factors. Additionally, the highly mobile nature of bat hosts may expose all members of the community to a wide and uniform range of environmental/dietary factors, thereby overriding subtle host effects.

ARGs and VFGs in bats respond to environmental changes over time, which are accompanied by physiological transformations in the bat gut during pregnancy and lactation. Previous studies have documented shifts in the gut bacterial composition across different reproductive states, potentially linked to dietary adaptations that meet evolving nutritional and energy demands^33,68^. The observed increase in insect prey consumption from pregnancy to lactation supports the hypothesis that bats may alter their diet to fulfill nutritional requirements. During this period, the expansion of the resistome, coupled with dietary changes, may pose a public health challenge by enlarging the environmental resistance gene pool and increasing the risk of infection in newborn bats through mother-to-infant vertical transmission^64^. Nevertheless, this expansion in dietary variety could also result from seasonal increases in insect activity. The influence of natural diets on gut bacteria, antibiotic resistome, and virulome is often attributed to alterations in the nutritional content of food^40,69–71^. Increased dietary protein may promote ARG diversity by inducing shifts in microbial composition^69,72^. Therefore, further research is needed to elucidate the relationship between specific dietary nutritional components, the gut microbiota, and the resistome in wild bats. Additionally, considering insects as indicators of environmental pollution, the increased insect diversity in the diet might expose bat guts to more environmental pollutants. The accumulation of pollutants within the food web and their association with dietary diversity has been observed in various bat species^73,74^. The strong correlation between dietary composition, antibiotics, and ARGs observed in this study highlights that diet-mediated antibiotic intake is a key driver of the resistome. As prey availability and habitat expand, the gut resistome of bats will increasingly be influenced by broader environmental factors beyond their feeding grounds. Although it is difficult to quantitatively describe the relationship between food composition and antibiotic residues due to the lack of quantification of antibiotics in individual insects, the core role of food composition is directly supported rather than inferred from multi-step correlations. In summary, diet serves as an intermediary between the gut and the external environment, playing a central role, alongside environmental factors, in shaping the assembly and evolution of the microbiome, resistome, and virulome.

## Methods

### Study design and sample collection

Adult bats were randomly captured from a breeding colony beneath an overpass in Acheng District, Harbin City, Heilongjiang Province, China (127°00’ E, 45°33’ N). To reduce the impact on bats, we used non-invasive fecal sampling instead of collecting intestinal tissue. Fecal samples do not fully represent the entire gut microbiota in studies of gut microecology^75^. However, for the purpose of examining gut microecology, public health risk, and bat protection, non-invasive sampling methods (i.e., fecal collection) are both necessary and reasonable^76^. Sampling was conducted across six time points to cover key reproductive stages of female bats, including pregnancy, parturition, lactation, and weaning periods of female bats. To assess age-related variations in gut microbiota, resistome, and virulome, we additionally captured 3–4-week-old subadults that had recently initiated weaning and independent foraging^67^. Additionally, to examine potential urban-rural environmental influences, we incorporated 12 bat fecal samples from two rural locations. Further details on bat behavior and sampling protocols are provided in Fig. 1 and Supplementary Text 1. Mist nets were strategically deployed near habitats to capture individuals returning from foraging. For each captured bat, we recorded sex, weight (g), forearm length (mm), length of epiphyseal gaps (mm), and reproductive status (pregnancy, lactation, or weaning). Age estimation was based on the epiphyseal gaps length of the fourth metacarpal-phalangeal joint and forearm measurements^77^. Following data collection, bats were temporarily housed in sterilized kraft paper bags to facilitate fecal collection. Fecal samples were homogenized in 2 mL cryogenic tubes, flash-frozen in liquid nitrogen, and then divided into two portion: one portion (0.1 g) for residual antibiotics analysis, and the other (0.05 g) for DNA extraction. All sample collection methods carried out in this study were approved by the Laboratory Animal Welfare and Ethics Committee of Northeast Normal University (approval number: NENU-W-2017–10). No bats were harmed or killed during the collection process. All were released in good health at their original roost in the crevices of bridges, immediately after the collection was completed. All animal experiments adhered to the ARRIVE guidelines and were conducted in accordance with the National Research Council’s Guide for the Care and Use of Laboratory Animals.

### Detection of antibiotic residues

To explore the potential selection pressures on gut microbiota, the concentrations of 26 antibiotics in lyophilized fecal samples were analyzed, including three tetracyclines, four beta-lactams, four macrolides, seven quinolones, one aminoglycoside, one amphenicol, and six sulfonamide drugs. Specifically, samples were pretreated, and a solid phase extraction (SPE) method was employed for extraction of antibiotics. The concentrations of these antibiotics were determined by high-performance liquid chromatography-tandem mass spectrometry (HPLC-MS/MS) following a previously reported protocol^78,79^. Method validation parameters, including limits of detection (LOD), limit of quantification (LOQ), relative standard deviation (RSD), and detailed extraction protocols are further described in Supplementary Text 2 and Supplementary Table 3.

### DNA extraction, PCR amplification, and sequencing

Genomic DNA was extracted from the fecal samples using the D4015-00Stool DNA Kit (Omega Bio-Tek) following the manufacturer’s instructions. DNA integrity was assessed through 1% agarose gel electrophoresis, while concentration and purity were determined using both a NanoDrop microvolume spectrophotometer and Qubit fluorometer (Thermo Fisher Scientific, USA). For diet analysis, we amplified a 225 bp fragment of the mitochondrial cytochrome oxidase I (COI) gene using the primer pair LCO-1490 (5′-GGTCAACAAATCATAAAGATATTGG-3′) and ZBJ-ArtR2c (5′-WACTAATCAATTWCCAAATCCTCC-3′)^80,81^. PCR amplification was performed following established protocols^82^. The resulting PCR products were purified, quantified, and subsequently sequenced on the Illumina Miseq platform (2 × 300 bp paired ends) by Majorbio BioPharm Technology Co., Ltd (Shanghai, China) following their standard operating procedures. For metagenomic library preparation, DNA samples were fragmented into approximately 300 bp pieces using a Covaris M220 Focused-ultrasonicator and processed using TruSeq DNA PCR-Free Library Preparation Kit (Illumina, San Diego, CA, USA). The quality of the metagenomic library was evaluated with a Qubit Fluorimeter. Then, the library was sequenced on the DNBSEQ-T7 platform (MGI, Shenzhen, China) using a paired-end 150 bp configuration, generating raw data of approximately 10 Gb and 72.78 million reads per sample.

### Diet analysis

After demultiplexing, the resulting COI gene sequences were quality filtered with Fastp v0.23.4^83^ and merged with FLASH v1.2.11^84^. Then the high-quality sequences were de-noised using DADA2^85^ plugin in the Qiime2 v2022.2^86^ pipeline with recommended parameters, resulting in amplicon sequence variants (ASVs). To minimize the effects of sequencing depth on alpha and beta diversity measure, the number of sequences from each sample was rarefied to the minimum number of sequences among all samples. For insect identification, taxonomic assignment of ASVs was performed using the Blast+ consensus taxonomy classifier implemented in Qiime2 and the NCBI_nt v20230830 database. The ASV set identified as Arthropoda was retained for subsequent analysis. The proportion of specific taxa assigned to insects in each sample was quantified as the food composition of bat individuals.

### Preprocessing of metagenomic data

The raw data from metagenomic sequencing was subjected to quality evaluation using Fastp, followed by quality control, adapter trimming, and quality filtering. Bowtie2 v2.3.5^87^ was employed to map reads to the reference genome, removing host contamination to obtain clean data. Since the reference genome of *V*. *sinensis* was unavailable in the public database, we used the genome of its closely related species^88^, *Vespertilio murinus* (GenBank accession: GCA_963924515.1), as a proxy. Using a closely related genome as a reference may lead to residual host contamination, which could introduce biases in downstream analyses. To minimize this, it is essential to align database sequences during the bacterial taxonomic annotation process to ensure data cleanliness. Under the ‘very sensitive’ alignment mode, less than 1% of the reads in each sample were removed as host contamination. Following this substitution, we performed an additional quality assessment on the cleaned sequencing data to verify the efficacy of the quality control steps. Supplementary Fig. 17 provides more details on the bioinformatics analysis of metagenomic data.

### Contig assembly and genome reconstruction

To maximize sequence utilization, we performed de novo co-assembly of the cleaned reads across all samples using MEGAHIT v1.2.9^89^. The resulting contigs was then quality-assessed with QUAST v5.2.0^90^, and only those exceeding 500 bp in length were retained for downstream analyses.

The metaWRAP v1.3.2^91^ pipeline was used to recover MAGs. Specifically, MetaBAT2^92^ and MaxBin2^93^ in the *binning* module were used to generate metagenomic bins from reserved contigs. The *bin_refinement* module was used to consolidate binning predictions to improve the quality of bins. The completeness and contamination of the recovered MAGs were assessed using CheckM v1.2.2^94^ with default parameters. All recovered MAGs were de-replicated using dRep v3.4.5^95^ with a 95% ANI threshold^96,97^, while medium-quality (completeness ≥ 50% and contamination ≤ 10%) and high-quality (completeness ≥ 90% and contamination ≤ 5%) draft MAGs were retained for downstream analysis^98^. The relative abundance of non-redundant MAGs was calculated using the *quant_bins* module, and taxonomic classification was conducted using GTDB-Tk v2.4.0^99^ and GTDB (release 220). The phylogenetic tree of bacteria was reconstructed in the *gtdb_infer* module and visualized using iTOL (https://itol.embl.de).

### Microbial taxonomy and functional annotation

The taxonomic annotation and abundance estimate of microorganisms were performed using the Kraken software suite^100^, including Kraken v2.1.3^101^ and Bracken v2.9^102^. Prodigal v2.6.3^103^ with ‘meta’ mode was used to predict open reading frames (ORFs) from filtered contigs. Cd-hit v4.8.1^104^ was used to cluster and deduplicate the predicted genes, while Salmon v1.10.1^105^ was used for gene quantification. The resulting non-redundant gene set was used for functional annotations. The eggNOG-mapper v2.1.12^106^ program was used to map amino acid sequences to the evolutionary genealogy of Genes Non-supervised Orthologies Groups (eggNOG) v5.0^107^ databases with pre-set parameter (e value ≤ 1e^−5^). Functional profile was constructed based on the Transcripts Per Million (TPM) value of genes annotated to each KEGG ortholog (KO) in each sample. To reveal specific pathways of response time variation, functional clustering and enrichment analyses were conducted using the *reporterscore* package based on Generalized Reporter Score-based Analysis (GRSA) method^108^.

### Read-based and contig-based identification of ARGs and VFGs

The ARGs-OAP v3.0^109^ pipeline designed for short reads and the structured database SARG v3.2.1-S with hierarchical annotations of ARG type, mechanism, subtype, and reference sequence were used to characterize the resistance profile. Each ARG was assessed for risk based on human-associated enrichment, gene mobility, and host pathogenicity, and classified into ranks Ⅰ, Ⅱ, Ⅲ, Ⅳ, and ‘notassessed’, with lower rank indicating higher risk^110^. To characterize the virulence factor genes (VFGs), the virulence factor database (VFDB)^111^ were integrated into the ARGs-OAP pipeline to provide standardized and multiple annotation levels of gene quantification.

To investigate the mobility and pathogenic risk of genes, flanks of ARG-like sequences and VFG-like sequences were assembled, and adjacent mobile genetic elements (MGEs) were annotated. Specifically, mobileOG-pl (https://github.com/clb21565/mobileOG-db/tree/main/mobileOG-pl) was used to align contigs with the mobileOG-db v1.6^112^ database. MobileOG-db is a database of protein families mediating the integration/excision, replication/recombination/repair, stability/defense, or transfer of bacterial mobile genetic elements and phages as well as the associated transcriptional regulators of these processes. To further estimate the mobility of ARGs and VFGs, the genetic location (plasmid/chromosome/unclassified) of ARG/VFG-carrying contigs was determined using PlasFlow v1.1^113^. Plascad v1.17^114^ was used to classify identified plasmids into conjugative, mobilizable or non-mobilizable, with Position-Specific Iterated BLAST used for mobilization typing (MOB) (MOB_C_: 1e^−4^, MOB_F_: 1e^−8^, MOB_H_: 1e^−2^, MOB_P_: 1e^−4^, MOB_Q_: 1e^−4^, MOB_V_: 1e^−2^, MOB_B_: 1e^−4^ and MOB_T_: 1e^−4^). The mobility of plasmids was further validated using MOBFinder v1.0^115^. Subsequently, ARG/VFG-carrying contigs were assigned taxonomically using Kraken v2.1.4^101^ to identify microbial hosts. A pseudo-phylogenetic tree was constructed to visualize the bacterial hosts of ARG-MGE-VFG contigs, namely MGE-carrying PARBs.

### Statistical analysis

To examine microbial community dynamics, we performed statistical analyses using *MicrobiomeStat* (https://github.com/cafferychen777/MicrobiomeStat) in R. Specifically, the alpha diversity metrics (Shannon index, Simpson index, and Chao1 index) for both bacterial communities and functional genes were calculated for each sample using the *vegan* package. The Kruskal-Wallis H test was employed for multiple group comparisons, while the Mann-Whitney U test was utilized for comparing two groups. To resolve the variation of the observed features, we conducted non-metric multidimensional scaling (NMDS) based on Bray-Curtis distance, with statistical significance evaluated via permutation multivariate analysis of variance (PERMANOVA) using the *adonis2* function. To identify microbial features distinguishing different groups, we applied a random forest (RF) method with 5,000 decision trees. Procrustes analysis was performed to explore potential consistency in dietary composition, antibiotic residues, bacterial community and resistance gene composition. Distance-based redundancy analysis (db-RDA) was implemented to further explore antibiotics that have a significant impact on ARG profile. Networks of bacteria, KEGG function, ARGs, and VFGs were constructed based on Spearman correlation and visualized in Gephi v0.10.1^116^. Additionally, simple linear correlations with FDR correction were calculated between the multivariate variables. To assess the relative contribution of deterministic and stochastic processes to the assembly of bacterial communities and resistome, neutral community models (NCM) were constructed and normalized stochasticity ratios (NSTs) were calculated based on null models. Finally, we used partial least squares path modeling (PLS-PM) to quantify how host traits (sex, age, body size, and reproductive status), environment factors (location and time), and diets (dietary composition, antibiotics residue) affect bacterial communities, ARGs, and VFGs.

## Supplementary information

Supplementary materials

## Data Availability

Sequence data that support the findings of this study have been deposited in the NCBI Sequence Read Archive (SRA) database with the primary accession code PRJNA1298781.

## Code availability

The underlying code used for ARG-VFG-MGE contigs identification in this study is available in Zenodo and can be accessed via this link [10.5281/zenodo.17628441].
