# Supplementary material for: Diet and environmental factors jointly drive the gut microbiome, resistome, and virulome of urban bats
Source: NPJ Biofilms Microbiomes. 2026 Feb 4;12:61. doi: 10.1038/s41522-026-00930-y (PMC12976136; doi:10.1038/s41522-026-00930-y)
Supplement: Supplementary file 1 — Supplementary materials [file 41522_2026_930_MOESM1_ESM.pdf]

**Supporting information to**

# Diet and environmental factors jointly drive the gut microbiome, resistome and virulome of urban bats

Long Huang[1], Ying-Ting Pu[1], Yan-Hui Zhao[1], Xiao-Yu Sun[1], Yue Zhu[1], Ya-Ping Lu[1], Hai-Xia Leng[1], Jiang Feng[1,2,4], Long-Ru Jin[1,3,*] & Ke-Ping Sun[1,2,*]

[1]Jilin Provincial Key Laboratory of Animal Resource Conservation and Utilization, Northeast Normal University, Changchun 130117, China

[2]Key Laboratory of Vegetation Ecology, Ministry of Education, Changchun 130024, China

[3]Jilin Engineering Laboratory for Avian Ecology and Conservation Genetics, School of Life Sciences, Northeast Normal University, Changchun 130024, China

[4]Jilin Provincial International Cooperation Key Laboratory for Biological Control of Agricultural Pests, Changchun 130118, China

[*]Correspondence: sunkp129@nenu.edu.cn and jinlr915@nenu.edu.cn.

Supplementary_materials:

**Supplementary Text 1. Overview of bat population and sampling sites.**

**Supplementary Text 2. Detection of antibiotic concentration.**

**Supplementary Table 1. The summary statistical information of bacterial MAGs.**

**Supplementary Table 2. The summary of inner model inferred by PLS-PM.**

**Supplementary Table 3. The multiple reaction monitoring (MRM) parameters for 26 antibiotics.**

**Supplementary Fig. 1 | Composition of the dominant (top 20) bacterial genera in each sample.**

**Supplementary Fig. 2 | Composition of the dominant (top 20) KEGG level 2 ortholog groups in each sample.**

**Supplementary Fig. 3 | The pseudo phylogenetic tree displays MGE-carrying pathogenic antibiotic-resistant bacteria.**

**Supplementary Fig. 4 | The diversity of antibiotic resistance genes (ARGs) and virulence factor genes (VFGs) in fecal samples from subadult and adult bats.**

**Supplementary Fig. 5 | The diversity of bacterial genera and KEGG level 2 functions in fecal samples from subadult and adult bats.**

**Supplementary Fig. 6 | The diversity of antibiotic resistance genes (ARGs) and virulence factor genes (VFGs) in fecal samples from female and male bats.**

**Supplementary Fig. 7 | The diversity of bacterial genera and KEGG level 2 functions in fecal samples from female and male bats.**

**Supplementary Fig. 8 | The diversity of Bacterial genus in fecal samples from three locations.**

**Supplementary Fig. 9 | The diversity of KEGG level 2 functions in fecal samples from three locations.**

**Supplementary Fig. 10 | Longitudinal changes in VFG profile over time.**

**Supplementary Fig. 11 | The changes in bacterial community structure over time.**

**Supplementary Fig. 12 | Bacterial genera with significant changes in abundance among six time points.**

**Supplementary Fig. 13 | The relationship between abundance and diversity of bacteria, KEGG functions, ARGs, and VFGs.**

**Supplementary Fig. 14 | Enrichment analysis of KEGG functions with specific abundance variation patterns from t1 to t6.**

**Supplementary Fig. 15 | Community assembly mechanism of ARGs and VFGs.**

**Supplementary Fig. 16 | The external model of partial least squares path modeling (PLS-PM) in this study.**

**Supplementary Fig. 17 | The bioinformatics pipeline for shotgun metagenomic data analysis in this study.**

**Supplementary Text 1. Overview of bat population and sampling sites.**

The division of bat reproductive stages is based on long-term field observations. The Urban sampling site is located under an overpass in Acheng District, Harbin City, Heilongjiang Province, China, surrounded by multiple densely populated residential areas, where the permanent population exceeds 500,000. The bat population sampled primarily consisted of mother and offspring groups, with a population size of over 5,000. The initial sampling (t1) took place in mid to late May, coinciding with the migration of adult female bats into the area. The second sampling (t2) occurred during the later stages of pregnancy, where pregnant bats were identified through abdominal palpation, and these bats were expected to give birth by the end of June. The third sampling (t3) was conducted in the second week of post-birth. Juvenile bats exhibit rapid development, reaching sizes comparable to adults within 3-4 weeks, and subsequently begin to fly and forage independently. During this phase, fecal samples were collected from both adult bats (t4) and subadults (Subadult_male and Subadult_female) transitioning from a breast milk diet to an insect diet. Sampling continued until late September, with two additional samplings (t5 and t6) of adult bats before their departure. The Rural_SY sampling site is located in the attic of a house in Sanjiazi Village, Shuangyang District, Changchun City, Jilin Province, China. This area is sparsely populated by farmers and is surrounded by corn and rice fields. The Rural_YJ sampling site is situated on the roof of an abandoned factory building in Toudao Town, Helong City, Yanbian Korean Autonomous Prefecture, Jilin Province, China. This area is close to the center of the village and town, with a permanent population of over 20,000 people. The straight-line distance between the three sampling points is approximately 300 km.

**Supplementary Text 2. Detection of antibiotic concentration.**

In this study, a total of 56 bat fecal samples were analyzed for antibiotic residues, while four additional samples could not be tested due to insufficient sample mass. The feces samples were pretreated, and solid-phase extraction was performed following a previously described method with appropriate modifications [1]. Specifically, lyophilized samples (0.1 g) were extracted with 10 mL of extraction solutions, which included 2.5 mL of methanol (Sigma-Aldrich, St. Louis, MO), 2.5 mL of acetonitrile, 0.02 g $Na_2$-EDTA, and 5 mL of McIllvaine buffer (Citrate-phosphate buffer, pH 4). The extract was vortexed at 3,000 rpm for 1 min, and then subjected to ultrasonication for 15 min. After centrifugation at 10,000 rpm for 10 min, approximately 10 mL of the supernatant was collected in a brown glass bottle. This process was repeated three times, combining the three supernatants to a final volume of approximately 30 mL. The final supernatant mixture was then blended and diluted to 100 mL with ultra-pure water, before proceeding with solid-phase extraction. The solid-phase extraction process was performed using a fully automatic solid-phase extraction system (Aqua Trace ASPE899, Shimadzu Corporation) with C18 cartridges (500 mg, 6 mL). The HPLC-MS/MS analysis was then conducted with an Agilent 1260 Infinity II-6470 HPLC (Agilent Technologies, Germany) equipped with an electrospray ionization (ESI) detector. Calibration curves were established by choosing seven concentration levels (0.005, 0.01, 0.05, 0.1, 0.2, 0.5, and 1 mg/L) for quantification, and the correlation coefficients ($R^2$) for all the antibiotics were typically above 0.99. Method recoveries for antibiotics, including six sulfonamides, were determined using three fecal samples spiked at gradient concentration levels (10 ng/L, 250 ng/L, and 500 ng/L). The recoveries of target compounds at these four spiked levels ranged from 64.7% to 99%. The relative standard deviations (RSD) were calculated from triplicate samples spiked at the same concentration, with RSD values ranging from 3.3% to 6.4%. The limit of quantification (LOQ) was determined as the lowest concentration yielding a signal to noise ratio (S/N) $\geq$ 10 and ranged from 1.2 to 5.6 ng/g.

1 Luo, Y. *et al.* Characteristics of wild bird resistomes and dissemination of antibiotic resistance genes in interconnected bird-habitat systems revealed by similarity of $bla_{TEM}$ polymorphic sequences. *Environ. Sci. Technol.* **56,** 15084-15095 (2022).

**Supplementary Table 1. The summary statistical information of bacterial MAGs.**

| Bin | Completeness (%) | Contamination (%) | Strain heterogeneity (%) | Closest_genome_reference | Closest_genome_ANI (%) |
|---|---|---|---|---|---|
| bin.104 | 63.79 | 2.59 | 50 | N/A | N/A |
| bin.105 | 91.03 | 4.55 | 75 | GCF_025985425.1 | 97.98 |
| bin.111 | 60.28 | 0.86 | 0 | N/A | N/A |
| bin.112 | 100 | 3.37 | 0 | N/A | N/A |
| bin.118 | 71.93 | 1.75 | 100 | GCF_007991875.1 | 98.01 |
| bin.12 | 86.05 | 0.55 | 0 | N/A | N/A |
| bin.13 | 78.24 | 7.76 | 85.71 | GCF_001742285.1 | 97.25 |
| bin.130 | 98.32 | 3.48 | 66.67 | GCF_025449335.1 | 96.59 |
| bin.134 | 93.45 | 8.33 | 12 | N/A | N/A |
| bin.138 | 92.13 | 3.05 | 14.29 | N/A | N/A |
| bin.142 | 90.55 | 6.33 | 95.45 | N/A | N/A |
| bin.144 | 82.44 | 1.55 | 40 | N/A | N/A |
| bin.148 | 96.44 | 9.75 | 27.5 | GCF_019218635.1 | 98.35 |
| bin.150 | 66.22 | 3.23 | 25 | N/A | N/A |
| bin.156 | 98.22 | 1.87 | 0 | N/A | N/A |
| bin.161 | 95.71 | 1.5 | 20 | N/A | N/A |
| bin.164 | 95.83 | 3.36 | 14.29 | N/A | N/A |
| bin.165 | 59.18 | 2.03 | 16.67 | N/A | N/A |
| bin.17 | 95.35 | 6.78 | 50 | N/A | N/A |
| bin.170 | 89.86 | 0.03 | 100 | N/A | N/A |
| bin.172 | 89.3 | 1.91 | 25 | N/A | N/A |
| bin.177 | 97.42 | 4.73 | 0 | N/A | N/A |
| bin.178 | 92.43 | 8.5 | 48.65 | N/A | N/A |
| bin.180 | 82.47 | 2.33 | 12.5 | N/A | N/A |
| bin.191 | 54.55 | 3.45 | 100 | GCF_000013285.1 | 96.66 |
| bin.192 | 97.36 | 2.8 | 0 | N/A | N/A |
| bin.194 | 93.02 | 0.78 | 0 | N/A | N/A |
| bin.196 | 91.01 | 4.49 | 25 | N/A | N/A |
| bin.20 | 92.52 | 6.34 | 16.67 | N/A | N/A |
| bin.201 | 99.09 | 2.97 | 100 | N/A | N/A |
| bin.204 | 57.03 | 3.3 | 26.67 | N/A | N/A |
| bin.208 | 87.98 | 4.43 | 6.25 | N/A | N/A |
| bin.214 | 58.23 | 3.45 | 100 | N/A | N/A |
| bin.215 | 86.12 | 2.54 | 0 | N/A | N/A |
| bin.216 | 64.22 | 0.14 | 0 | N/A | N/A |
| bin.222 | 98.92 | 0 | 0 | GCF_004014855.1 | 98.92 |
| bin.224 | 95.68 | 0.31 | 100 | GCF_000146325.1 | 97.16 |
| bin.227 | 59.48 | 8.62 | 85.71 | GCF_000710035.2 | 98.73 |
| bin.228 | 87.8 | 2.26 | 20 | N/A | N/A |
| bin.229 | 85.18 | 7.34 | 52.17 | N/A | N/A |

| | | | | | |
|---|---|---|---|---|---|
| bin.23 | 51.06 | 0 | 0 | N/A | N/A |
| bin.230 | 94.93 | 1.35 | 0 | N/A | N/A |
| bin.237 | 95.75 | 9.06 | 84 | GCF_014634745.1 | 98.2 |
| bin.240 | 59.63 | 7.62 | 4.35 | N/A | N/A |
| bin.244 | 82.96 | 1.53 | 75 | GCF_001294465.1 | 99.56 |
| bin.246 | 95.17 | 5.03 | 18.18 | N/A | N/A |
| bin.247 | 86.8 | 6.41 | 60.53 | GCF_024169515.1 | 97.93 |
| bin.266 | 97.02 | 3.42 | 37.5 | N/A | N/A |
| bin.269 | 91.56 | 2.68 | 63.64 | N/A | N/A |
| bin.27 | 97.54 | 1.26 | 58.33 | N/A | N/A |
| bin.272 | 59.48 | 1.72 | 100 | GCF_000407445.1 | 97.7 |
| bin.280 | 88.57 | 0 | 0 | GCF_018437265.1 | 97.86 |
| bin.281 | 89.47 | 4.03 | 45 | N/A | N/A |
| bin.290 | 91.13 | 9.76 | 5.88 | GCF_900091705.1 | 96.32 |
| bin.292 | 97.27 | 4.19 | 7.69 | N/A | N/A |
| bin.296 | 98.24 | 0.49 | 0 | N/A | N/A |
| bin.297 | 69.83 | 5.17 | 100 | N/A | N/A |
| bin.299 | 90.35 | 8.57 | 60.53 | N/A | N/A |
| bin.30 | 90.55 | 3.76 | 57.14 | GCF_002050515.1 | 99.01 |
| bin.301 | 52.51 | 6.12 | 16.22 | N/A | N/A |
| bin.304 | 89.15 | 8.58 | 63.64 | GCF_030814225.1 | 95.67 |
| bin.305 | 54.94 | 3.45 | 100 | GCF_001975225.1 | 97.39 |
| bin.306 | 92.77 | 4.28 | 57.89 | GCF_003991975.1 | 96.35 |
| bin.309 | 98.64 | 0 | 0 | N/A | N/A |
| bin.31 | 98.36 | 1.79 | 33.33 | N/A | N/A |
| bin.310 | 90.32 | 1.58 | 7.14 | N/A | N/A |
| bin.312 | 62.07 | 0.86 | 100 | GCF_000730565.1 | 99.33 |
| bin.313 | 96.12 | 3.43 | 50 | GCF_000193635.1 | 99.86 |
| bin.318 | 88.31 | 9.84 | 77.78 | GCF_900087055.1 | 97.12 |
| bin.319 | 82.49 | 4.64 | 43.24 | N/A | N/A |
| bin.32 | 96.76 | 4.61 | 85.71 | N/A | N/A |
| bin.322 | 70.01 | 2.3 | 63.64 | N/A | N/A |
| bin.324 | 59.87 | 3.71 | 8.33 | N/A | N/A |
| bin.327 | 72.81 | 5.36 | 3.23 | N/A | N/A |
| bin.328 | 86.71 | 4.49 | 35.29 | GCF_017830045.1 | 98.84 |
| bin.330 | 95.99 | 5.52 | 28.57 | N/A | N/A |
| bin.331 | 92.67 | 4.65 | 52.54 | GCF_003610915.1 | 97.65 |
| bin.332 | 52.98 | 5.26 | 100 | GCA_016618195.1 | 99.5 |
| bin.334 | 63.4 | 6.9 | 80 | N/A | N/A |
| bin.335 | 57.93 | 3.45 | 0 | N/A | N/A |
| bin.339 | 96.17 | 5.87 | 53.33 | N/A | N/A |
| bin.340 | 92.76 | 7.59 | 88.89 | GCF_001436605.1 | 98.65 |
| bin.353 | 93.51 | 8.87 | 27.27 | N/A | N/A |
| bin.355 | 94.95 | 8.68 | 45 | N/A | N/A |

| | | | | | |
|---|---|---|---|---|---|
| bin.358 | 50.31 | 0 | 0 | N/A | N/A |
| bin.363 | 85 | 7.04 | 52 | N/A | N/A |
| bin.364 | 82.26 | 0.23 | 50 | N/A | N/A |
| bin.366 | 51.8 | 3.45 | 100 | GCF_018494055.1 | 98.12 |
| bin.371 | 97.12 | 3.57 | 100 | N/A | N/A |
| bin.373 | 76.9 | 1.72 | 0 | N/A | N/A |
| bin.374 | 52.39 | 1.75 | 0 | GCF_000173415.1 | 99.09 |
| bin.375 | 88.43 | 1.44 | 28.57 | N/A | N/A |
| bin.377 | 91.71 | 1.13 | 0 | N/A | N/A |
| bin.38 | 61.33 | 6.6 | 57.14 | N/A | N/A |
| bin.389 | 97.89 | 3.5 | 33.33 | N/A | N/A |
| bin.39 | 98.17 | 3.13 | 53.85 | GCF_004117915.1 | 98.26 |
| bin.392 | 77.29 | 7.84 | 0 | N/A | N/A |
| bin.394 | 54.68 | 5.65 | 7.69 | N/A | N/A |
| bin.395 | 56.19 | 1.72 | 0 | GCF_000735455.1 | 98.95 |
| bin.396 | 97.68 | 0.4 | 0 | GCF_004367745.1 | 97.64 |
| bin.397 | 82.68 | 7.95 | 62.86 | N/A | N/A |
| bin.408 | 79.82 | 3.02 | 7.14 | N/A | N/A |
| bin.41 | 64.01 | 0.35 | 0 | N/A | N/A |
| bin.420 | 61.2 | 2.45 | 25 | GCF_002563895.1 | 98.96 |
| bin.421 | 94.97 | 1.26 | 44.44 | GCF_947648125.1 | 99.96 |
| bin.423 | 95.25 | 0 | 0 | N/A | N/A |
| bin.425 | 84.54 | 6.99 | 22.58 | N/A | N/A |
| bin.426 | 92.75 | 3.82 | 33.33 | N/A | N/A |
| bin.431 | 97.64 | 4.68 | 50 | GCF_000024405.1 | 98.34 |
| bin.433 | 65.79 | 0 | 0 | N/A | N/A |
| bin.435 | 97.35 | 2.4 | 80 | N/A | N/A |
| bin.446 | 99.52 | 2.39 | 28.57 | N/A | N/A |
| bin.448 | 51.44 | 4.31 | 66.67 | GCF_029024465.1 | 97.68 |
| bin.456 | 83.1 | 9.68 | 6.25 | GCF_014635045.1 | 99.65 |
| bin.457 | 91.77 | 1.03 | 50 | N/A | N/A |
| bin.460 | 86.52 | 3.08 | 41.94 | GCF_001922585.1 | 99.73 |
| bin.462 | 89.58 | 0.96 | 0 | N/A | N/A |
| bin.464 | 90 | 6.18 | 21.74 | N/A | N/A |
| bin.470 | 67.73 | 0.4 | 0 | N/A | N/A |
| bin.473 | 91.58 | 4.87 | 47.83 | N/A | N/A |
| bin.475 | 62.38 | 9.48 | 100 | GCF_014306135.1 | 95.17 |
| bin.478 | 91.61 | 2.8 | 0 | N/A | N/A |
| bin.48 | 93.45 | 1.14 | 50 | N/A | N/A |
| bin.481 | 96.37 | 5.95 | 18.18 | GCF_001654855.1 | 99.41 |
| bin.483 | 84.26 | 7.34 | 57.14 | GCF_012922555.1 | 97.61 |
| bin.489 | 57.4 | 0 | 0 | GCF_001656035.1 | 99.52 |
| bin.491 | 52.38 | 0.86 | 100 | N/A | N/A |
| bin.492 | 66.38 | 6.9 | 75 | GCF_028048155.1 | 98.22 |

| | | | | | |
|---|---|---|---|---|---|
| bin.50 | 95.26 | 1.18 | 66.67 | GCF_000012385.1 | 96.76 |
| bin.507 | 53.51 | 6.14 | 75 | GCF_027595165.1 | 97.56 |
| bin.513 | 89.59 | 2.7 | 25 | N/A | N/A |
| bin.519 | 79.35 | 1.48 | 60 | GCF_001438695.1 | 99.17 |
| bin.52 | 96.85 | 1.24 | 0 | N/A | N/A |
| bin.521 | 61.4 | 4.59 | 25 | N/A | N/A |
| bin.523 | 94.71 | 3.67 | 33.33 | N/A | N/A |
| bin.524 | 92.97 | 4.42 | 0 | N/A | N/A |
| bin.525 | 95.39 | 3.28 | 0 | N/A | N/A |
| bin.527 | 91.87 | 2.61 | 33.33 | N/A | N/A |
| bin.530 | 95.32 | 4.48 | 0 | N/A | N/A |
| bin.531 | 86.97 | 3.95 | 0 | N/A | N/A |
| bin.532 | 89.41 | 0.98 | 75 | GCF_000429565.1 | 99.43 |
| bin.535 | 96.74 | 0.62 | 0 | N/A | N/A |
| bin.537 | 70.11 | 5.86 | 29.63 | N/A | N/A |
| bin.538 | 56.54 | 6.9 | 66.67 | GCF_001655005.1 | 99.07 |
| bin.539 | 85.47 | 5.38 | 0 | N/A | N/A |
| bin.548 | 93.96 | 4.53 | 42.86 | GCF_001544215.1 | 98.19 |
| bin.549 | 54.31 | 0 | 0 | N/A | N/A |
| bin.550 | 93.91 | 1.21 | 33.33 | N/A | N/A |
| bin.551 | 64.62 | 3.55 | 55.56 | N/A | N/A |
| bin.555 | 61.22 | 1.79 | 71.43 | N/A | N/A |
| bin.556 | 62.5 | 1.27 | 12.5 | N/A | N/A |
| bin.557 | 94.92 | 8.27 | 9.52 | N/A | N/A |
| bin.56 | 78.43 | 5.27 | 27.78 | N/A | N/A |
| bin.564 | 93.97 | 3.25 | 100 | N/A | N/A |
| bin.565 | 80.24 | 0 | 0 | N/A | N/A |
| bin.567 | 86.6 | 2.99 | 100 | N/A | N/A |
| bin.568 | 69.77 | 2.14 | 37.5 | N/A | N/A |
| bin.569 | 65.52 | 3.45 | 100 | N/A | N/A |
| bin.57 | 94.23 | 2.11 | 42.86 | N/A | N/A |
| bin.574 | 98.28 | 0 | 0 | N/A | N/A |
| bin.578 | 58.62 | 1.72 | 0 | N/A | N/A |
| bin.579 | 75.44 | 2.52 | 75 | N/A | N/A |
| bin.58 | 95.26 | 3.34 | 0 | N/A | N/A |
| bin.581 | 93.93 | 5.23 | 55.56 | GCF_947250645.1 | 97.43 |
| bin.585 | 61.36 | 6.9 | 40 | N/A | N/A |
| bin.588 | 96.55 | 3.45 | 8.33 | GCF_029023865.1 | 98.79 |
| bin.592 | 88.87 | 3.55 | 100 | GCF_026625865.1 | 98.54 |
| bin.593 | 91.8 | 1.7 | 44.44 | GCA_913775305.1 | 99.44 |
| bin.598 | 87.65 | 9.8 | 80.3 | N/A | N/A |
| bin.60 | 98.67 | 0.6 | 50 | N/A | N/A |
| bin.603 | 86.9 | 0.81 | 0 | N/A | N/A |
| bin.604 | 74.09 | 2.95 | 54.55 | N/A | N/A |

| | | | | | |
|---|---|---|---|---|---|
| bin.608 | 60.67 | 1.12 | 0 | N/A | N/A |
| bin.61 | 68.37 | 7.06 | 72.22 | N/A | N/A |
| bin.611 | 55.99 | 0.81 | 100 | N/A | N/A |
| bin.614 | 95.03 | 2.83 | 85.71 | N/A | N/A |
| bin.615 | 85.82 | 0.88 | 0 | N/A | N/A |
| bin.616 | 93.08 | 5.02 | 43.48 | N/A | N/A |
| bin.618 | 74.93 | 1.03 | 50 | N/A | N/A |
| bin.626 | 96.9 | 0.85 | 50 | N/A | N/A |
| bin.629 | 69.18 | 4.82 | 4.76 | N/A | N/A |
| bin.631 | 53.09 | 1.72 | 100 | GCF_022647325.1 | 98.52 |
| bin.638 | 51.72 | 6.9 | 100 | N/A | N/A |
| bin.640 | 94.97 | 1.51 | 14.29 | GCF_013449735.1 | 98.36 |
| bin.642 | 86.03 | 4.17 | 60 | N/A | N/A |
| bin.643 | 98.89 | 4.74 | 16.67 | GCF_000744945.1 | 98.35 |
| bin.648 | 91.39 | 4.33 | 9.09 | N/A | N/A |
| bin.649 | 85.26 | 4.23 | 11.11 | N/A | N/A |
| bin.650 | 63.34 | 1.05 | 66.67 | N/A | N/A |
| bin.654 | 97.13 | 3.08 | 0 | N/A | N/A |
| bin.655 | 55.03 | 0 | 0 | N/A | N/A |
| bin.656 | 55.8 | 4.47 | 60 | N/A | N/A |
| bin.659 | 96.5 | 0.2 | 100 | N/A | N/A |
| bin.660 | 97.23 | 0.66 | 25 | N/A | N/A |
| bin.662 | 82.77 | 0 | 0 | N/A | N/A |
| bin.67 | 53.76 | 0 | 0 | N/A | N/A |
| bin.670 | 95.93 | 2.55 | 88.89 | GCF_013394305.1 | 99.41 |
| bin.674 | 64.15 | 0.51 | 20 | N/A | N/A |
| bin.676 | 92.43 | 7.21 | 52.63 | GCF_029024925.1 | 98.8 |
| bin.677 | 83.46 | 8.91 | 44.74 | GCF_005405485.1 | 97.38 |
| bin.684 | 99.5 | 1.66 | 50 | N/A | N/A |
| bin.685 | 80.56 | 8.36 | 33.33 | N/A | N/A |
| bin.693 | 91.38 | 4.08 | 12.5 | GCF_947646745.1 | 99.93 |
| bin.695 | 61.55 | 1.72 | 100 | N/A | N/A |
| bin.697 | 56.03 | 1.72 | 100 | N/A | N/A |
| bin.702 | 79.48 | 4.31 | 66.67 | N/A | N/A |
| bin.703 | 64.83 | 4.31 | 100 | GCF_000953675.1 | 97.84 |
| bin.706 | 82.73 | 3.72 | 81.82 | N/A | N/A |
| bin.708 | 56.79 | 4.49 | 0 | N/A | N/A |
| bin.709 | 58.51 | 1.27 | 0 | N/A | N/A |
| bin.710 | 81.74 | 4.54 | 91.67 | N/A | N/A |
| bin.713 | 74.26 | 5.17 | 12.5 | N/A | N/A |
| bin.72 | 92.13 | 0 | 0 | N/A | N/A |
| bin.77 | 88.67 | 0.43 | 100 | N/A | N/A |
| bin.78 | 84.04 | 4.31 | 20 | N/A | N/A |
| bin.79 | 79.71 | 4.27 | 84.62 | GCF_001891125.1 | 97.53 |

| | | | | | |
|---|---|---|---|---|---|
| bin.80 | 93.32 | 1.77 | 80 | N/A | N/A |
| bin.86 | 93.14 | 2.49 | 83.33 | N/A | N/A |
| bin.88 | 54.4 | 0.69 | 100 | GCF_002026305.1 | 97.51 |
| bin.90 | 81.46 | 2.72 | 0 | N/A | N/A |
| bin.97 | 96 | 0.24 | 100 | N/A | N/A |
| bin.99 | 65.49 | 4.24 | 33.33 | N/A | N/A |

114

**Supplementary Table 2. The summary of inner model inferred by PLS-PM.**

| | Type | $R^2$ | Block_Communality | Mean_Redundancy | AVE |
|---|---|---|---|---|---|
| Host_traits | Exogenous | 0 | 0.345 | 0 | 0.345 |
| Environment | Exogenous | 0 | 0.846 | 0 | 0.846 |
| Diet | Endogenous | 0.0901 | 0.552 | 0.0497 | 0.552 |
| Bacteria | Endogenous | 0.3042 | 0.489 | 0.1488 | 0.489 |
| ARG | Endogenous | 0.4011 | 0.528 | 0.2118 | 0.528 |
| VFG | Endogenous | 0.5672 | 0.627 | 0.3558 | 0.627 |

**Supplementary Table 3. The multiple reaction monitoring (MRM) parameters for 26 antibiotics.**

| Type | Compound | Abbreviation | Precursor ion | Product ion | Cone voltage (V) | Collision energy (V) |
|---|---|---|---|---|---|---|
| Sulfonamide | Sulfadiazine | SDZ | 251.1 | 156 | 100 | 8 |
| | Sulfamethoxazole | SMZ | 254.1 | 156 | 108 | 12 |
| | Sulfachloropyridazine | SCP | 285 | 156 | 108 | 12 |
| | Sulfadimidine | SDM | 279.1 | 186.1 | 116 | 12 |
| | Trimethoprim | TMP | 291.2 | 261.1 | 151 | 24 |
| | Sulfapyridine | SP | 250.1 | 156 | 150 | 17 |
| Tetracycline | Tetracycline | TET | 445.2 | 410.1 | 115 | 17 |
| | Oxytetracycline | OXY | 461.2 | 205.2 | 90 | 35 |
| | Doxycycline | DOX | 445.2 | 428.1 | 113 | 16 |
| Macrolide | Roxithromycin | ROX | 837.5 | 679.4 | 150 | 20 |
| | Erythromycin | ERY | 734.5 | 576.2 | 172 | 6 |
| | Clarithromycin | CLR | 748.5 | 590.4 | 100 | 18 |
| | Azithromycin | AZI | 749.5 | 591.4 | 90 | 30 |
| Quinolone | Enrofloxacin | ENR | 360.2 | 342.2 | 156 | 20 |
| | Lomefloxacin | LOM | 352.2 | 334 | 159 | 20 |
| | Norfloxacin | NOR | 320.1 | 302.1 | 150 | 25 |
| | Ciprofloxacin | CIP | 332.1 | 314 | 149 | 20 |
| | Ofloxacin | OFX | 362.2 | 318.2 | 149 | 20 |
| | Sparfloxacin | SPX | 393.2 | 375.2 | 164 | 20 |
| | Moxifloxacin | MXF | 402.2 | 384.2 | 150 | 20 |
| β-lactam | Ampicillin | AMP | 350.2 | 160 | 30 | 12 |
| | Cefalexin | CEL | 348.23 | 174.1 | 16 | 16 |
| | Amoxicillin | AMX | 366.2 | 349.1 | 27 | 8 |
| | Cefoxitin | FOX | 445.3 | 367 | 100 | 6 |
| Aminoglycoside | Spectinomycin dihydrochloride | SPD | 333.3 | 189.2 | 115 | 32 |
| Amphenicol | Florfenicol | FFC | 356 | 336 | 141 | 12 |

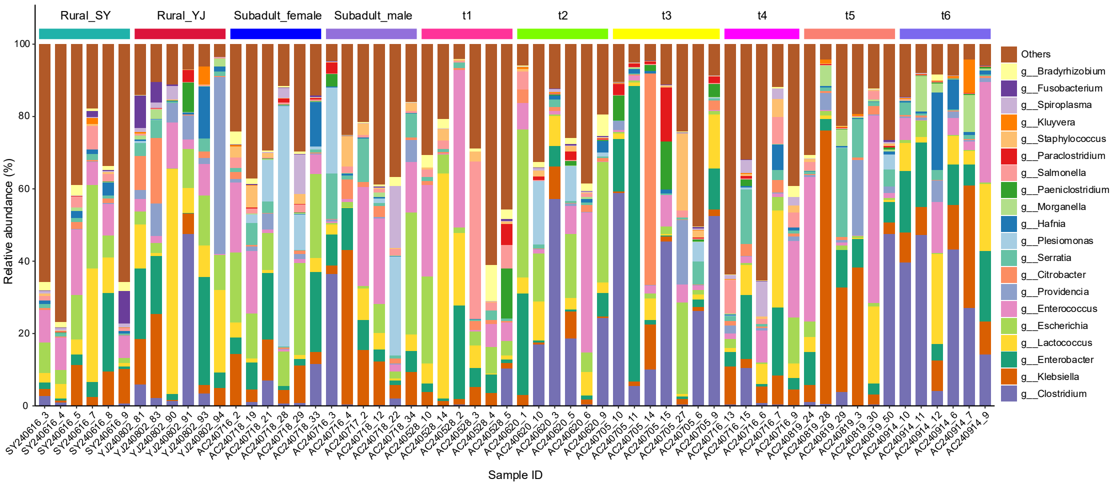


**Supplementary Fig. 1 | Composition of the dominant (top 20) bacterial genera in each sample.**

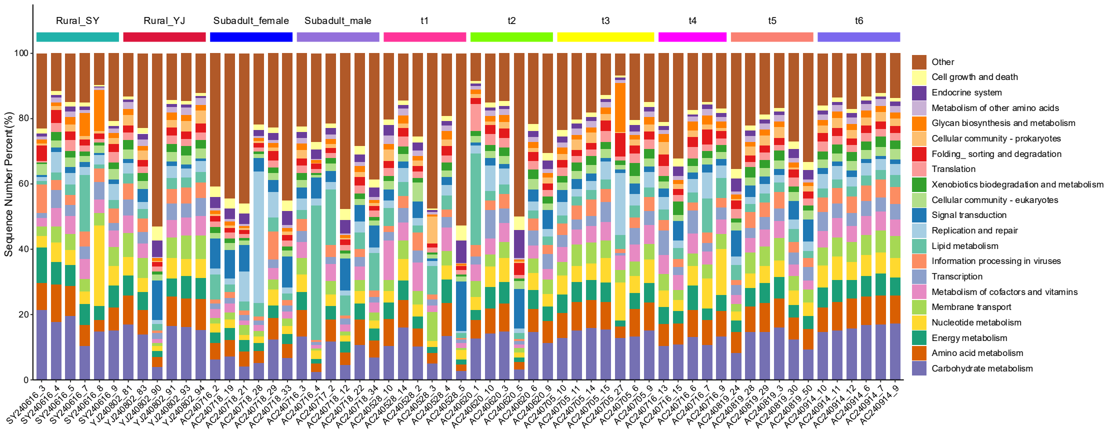


**Supplementary Fig. 2 | Composition of the dominant (top 20) KEGG level 2 ortholog groups in each sample.**

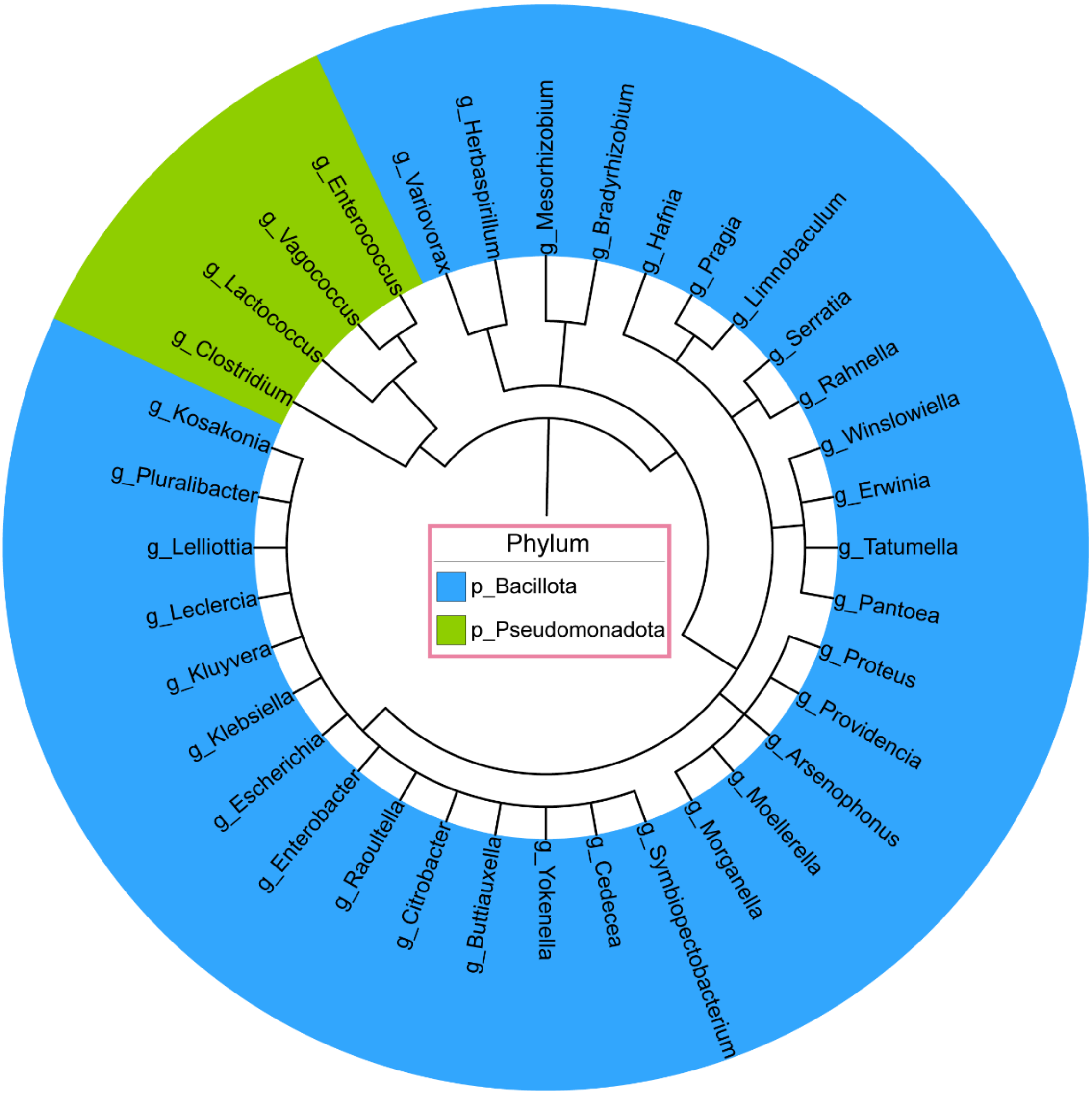


**Supplementary Fig. 3 | The pseudo phylogenetic tree displays MGE-carrying pathogenic antibiotic-resistant bacteria.**

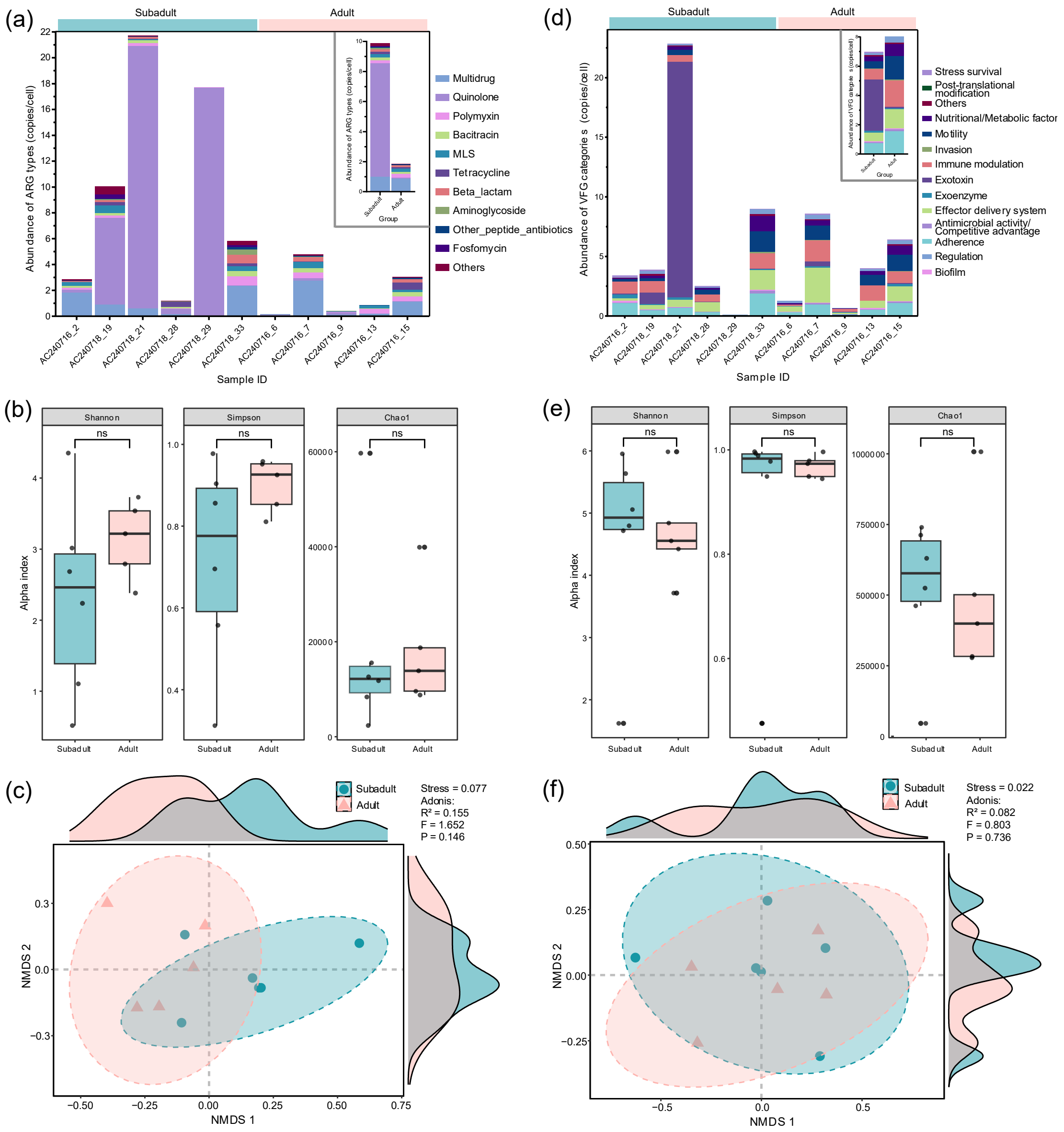


**Supplementary Fig. 4 | The diversity of antibiotic resistance genes (ARGs) and virulence factor genes (VFGs) in fecal samples from subadult and adult bats.** The stacked bar chart displays the dominant composition of ARG types **(a)** and VFG categories **(d)** in each sample from subadult and adult bats. The box plot displays the differences in alpha diversity of ARGs **(b)** and VFGs **(e)** among two groups. Non-metric multidimensional scaling (NMDS) ordinations with density maps reveal the differences in the community structure of ARGs **(c)** and VFGs **(f)**.

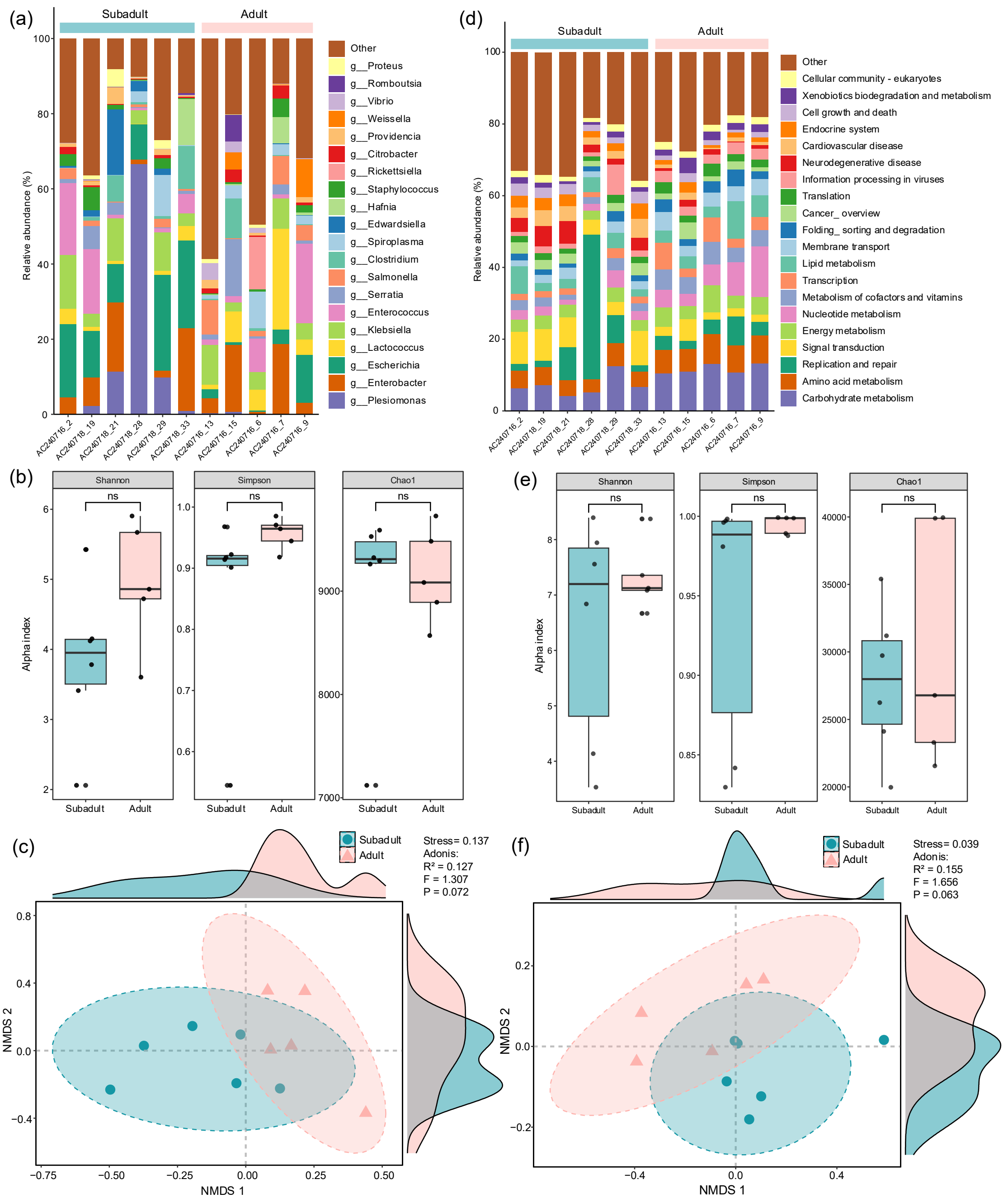


**Supplementary Fig. 5 | The diversity of bacterial genera and KEGG level 2 functions in fecal samples from subadult and adult bats.** The stacked bar chart displays the dominant composition of bacterial genera **(a)** and KEGG functions **(d)** in each sample from subadult and adult bats. The box plot displays the differences in alpha diversity of bacterial genera **(b)** and KEGG functions **(e)** among two groups. Non-metric multidimensional scaling (NMDS) ordinations with density maps reveal the differences in the community structure of bacteria **(c)** and KEGG functions **(f)**.

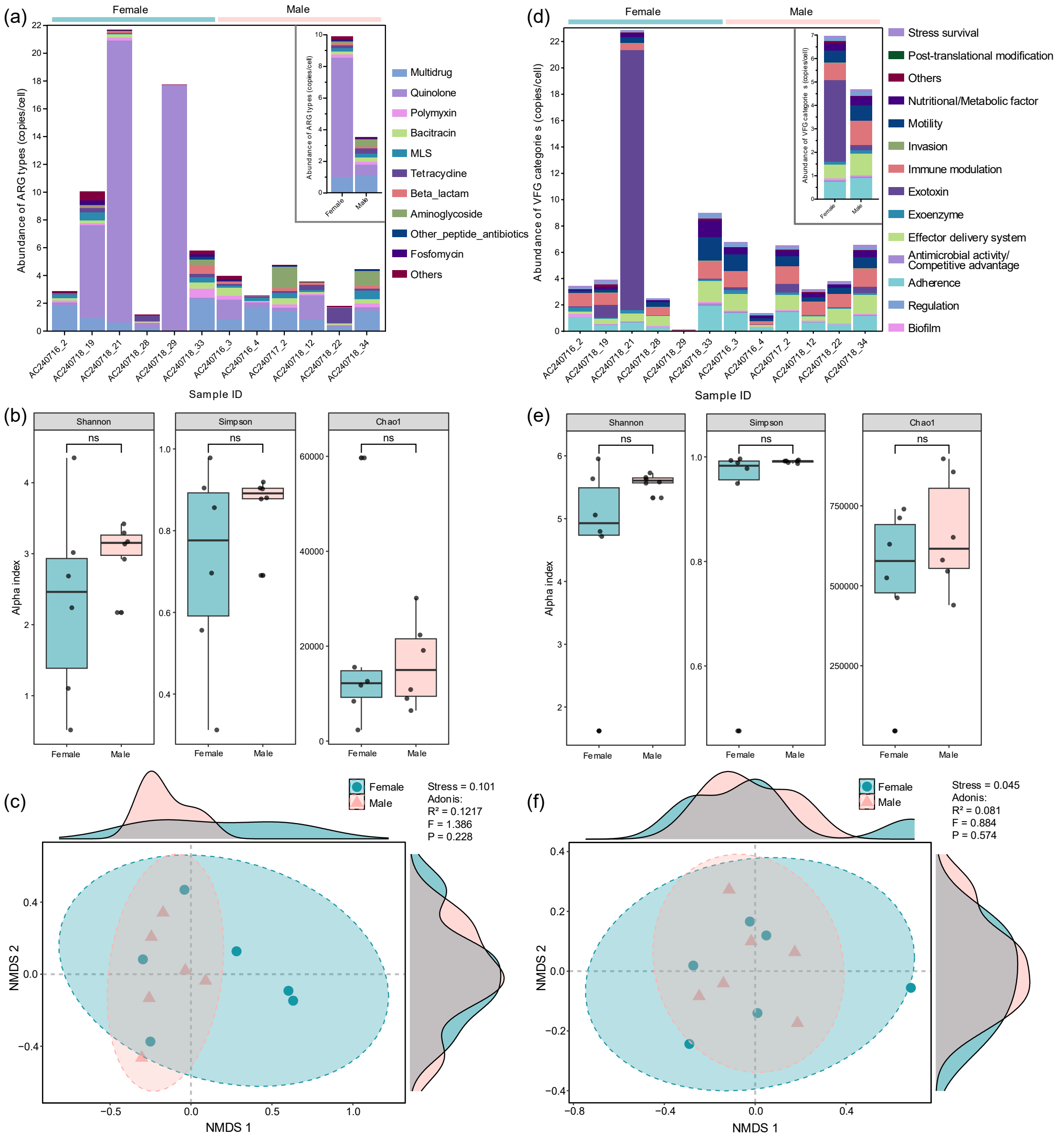


**Supplementary Fig. 6 | The diversity of antibiotic resistance genes (ARGs) and virulence factor genes (VFGs) in fecal samples from female and male bats.** The stacked bar chart displays the dominant composition of ARG types **(a)** and VFG categories **(d)** in each sample from female and male bats. The box plot displays the differences in alpha diversity of ARGs **(b)** and VFGs **(e)** among two groups. Non-metric multidimensional scaling (NMDS) ordinations with density maps reveal the differences in the community structure of ARGs **(c)** and VFGs **(f)**.

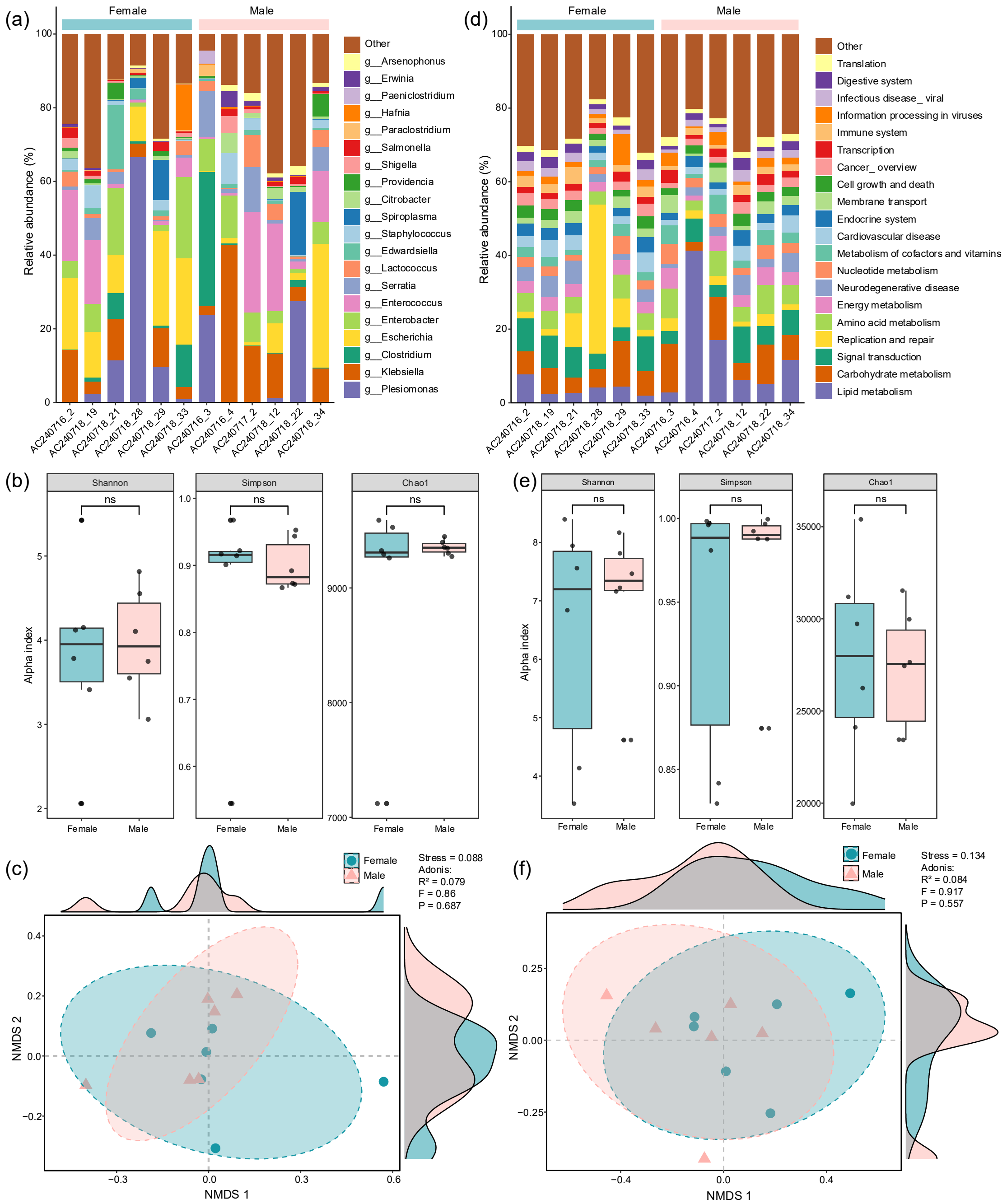


**Supplementary Fig. 7 | The diversity of bacterial genera and KEGG level 2 functions in fecal samples from female and male bats.** The stacked bar chart displays the dominant composition of bacterial genera **(a)** and KEGG functions **(d)** in each sample from female and male bats. The box plot displays the differences in alpha diversity of bacterial genera **(b)** and KEGG functions **(e)** among two groups. Non-metric multidimensional scaling (NMDS) ordinations with density maps reveal the differences in the community structure of bacteria **(c)** and KEGG functions **(f)**.

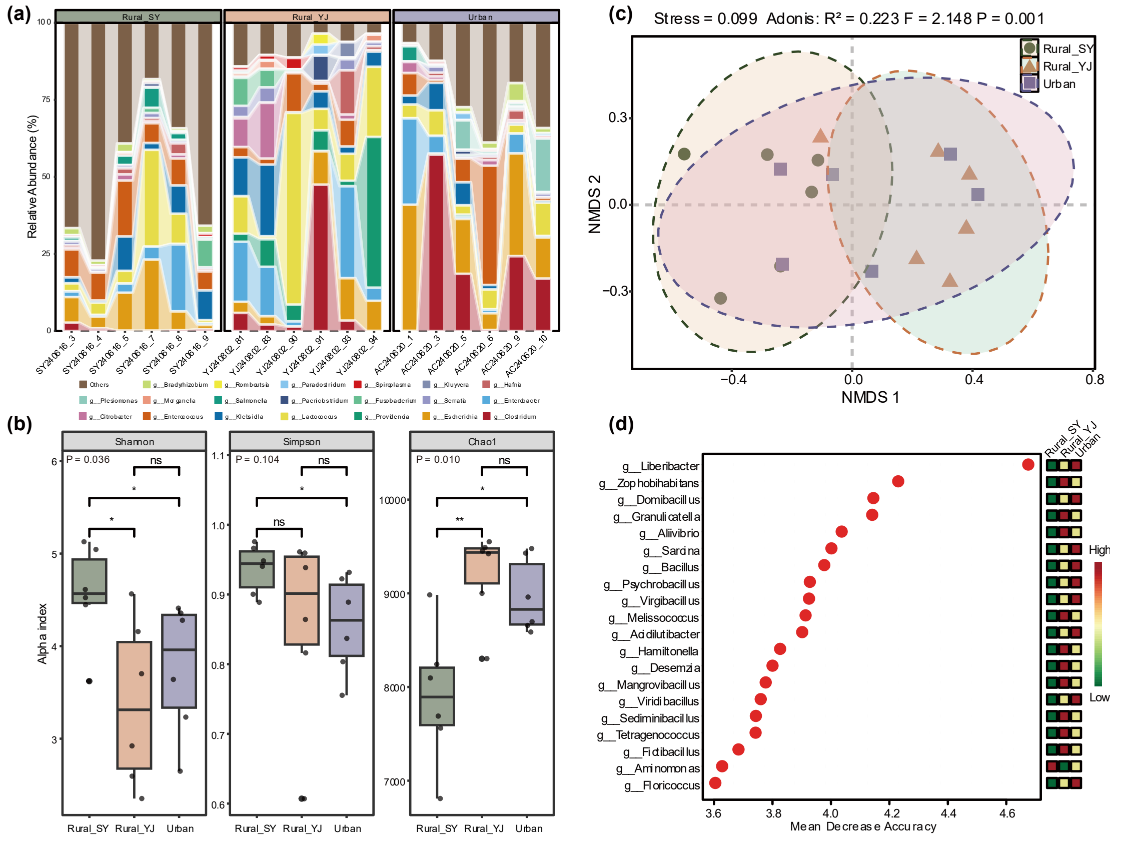


**Supplementary Fig. 8 | The diversity of Bacterial genus in fecal samples from three locations.** **(a)** The stacked bar chart displays the dominant composition of bacterial genus in each sample from three locations. **(b)** The box plot displays the differences in alpha diversity of bacterial genus among three groups. **(c)** Non-metric multidimensional scaling (NMDS) ordinations reveal the differences of bacterial community structure. **(d)** Random forest analysis identifies the main bacterial genera that significantly contributes to community differences. Distinct color blocks represent the average abundance of each feature.

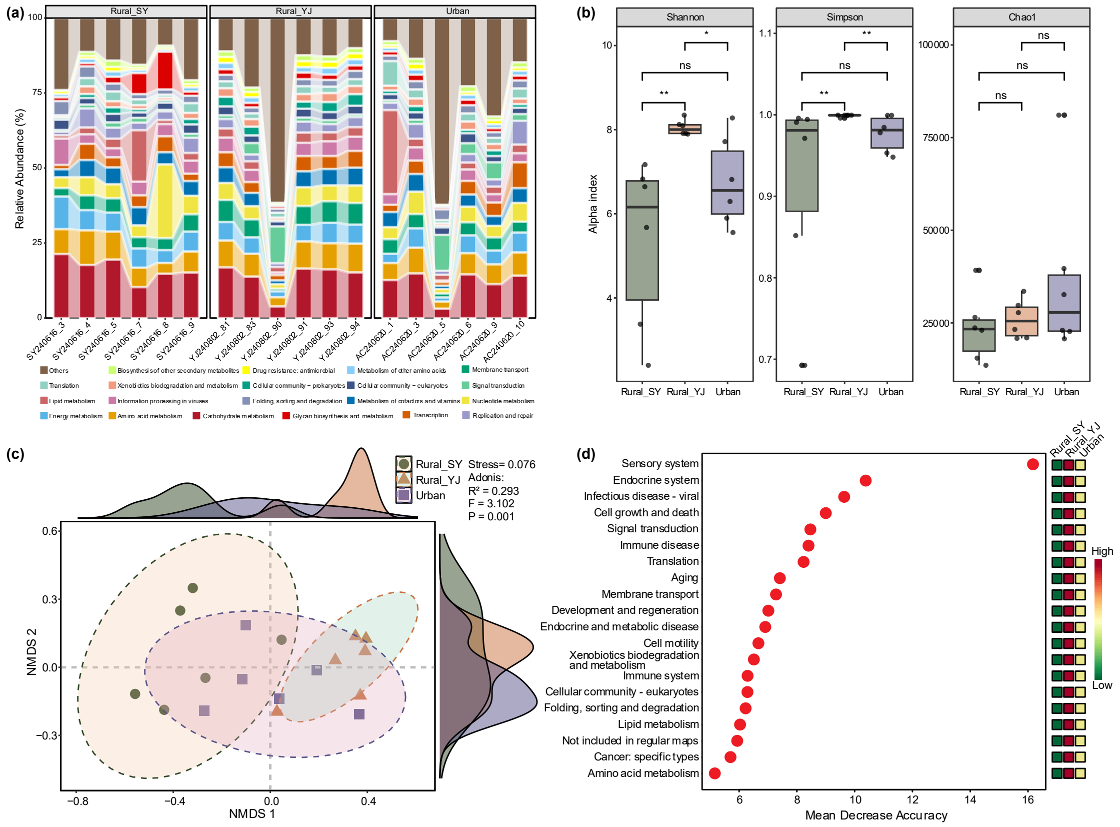


**Supplementary Fig. 9 | The diversity of KEGG level 2 functions in fecal samples from three locations. (a)** The stacked bar chart displays the dominant composition of KEGG functions in each sample from three locations. **(b)** The box plot displays the differences in alpha diversity of KEGG functions among three groups. **(c)** Non-metric multidimensional scaling (NMDS) ordinations with density maps reveal the differences of KEGG functions. **(d)** Random forest analysis identifies KEGG functions that significantly contribute to community differences. Distinct color blocks represent the average abundance of each feature.

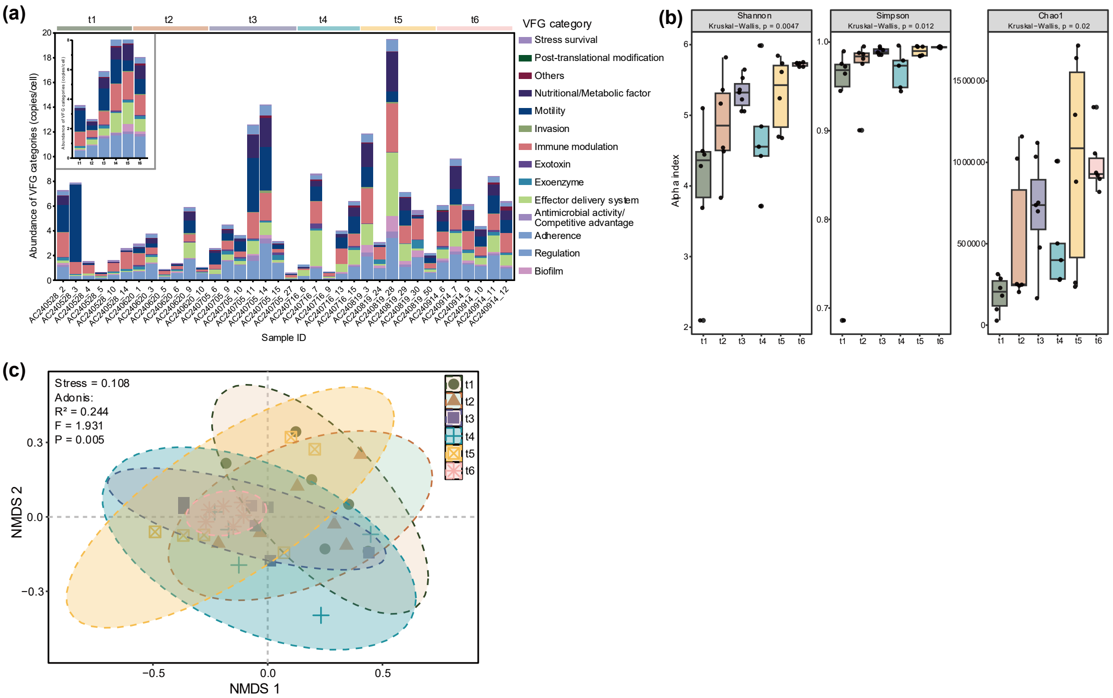


**Supplementary Fig. 10 | Longitudinal changes in VFG profile over time. (a)** The stacked bar chart displays the dominant composition of VFG categories in each sample from six time points. **(b)** The box plot displays the differences in alpha diversity of VFGs among six time points. **(c)** Non-metric multidimensional scaling (NMDS) ordinations reveal the differences of VFG profile among six time points.

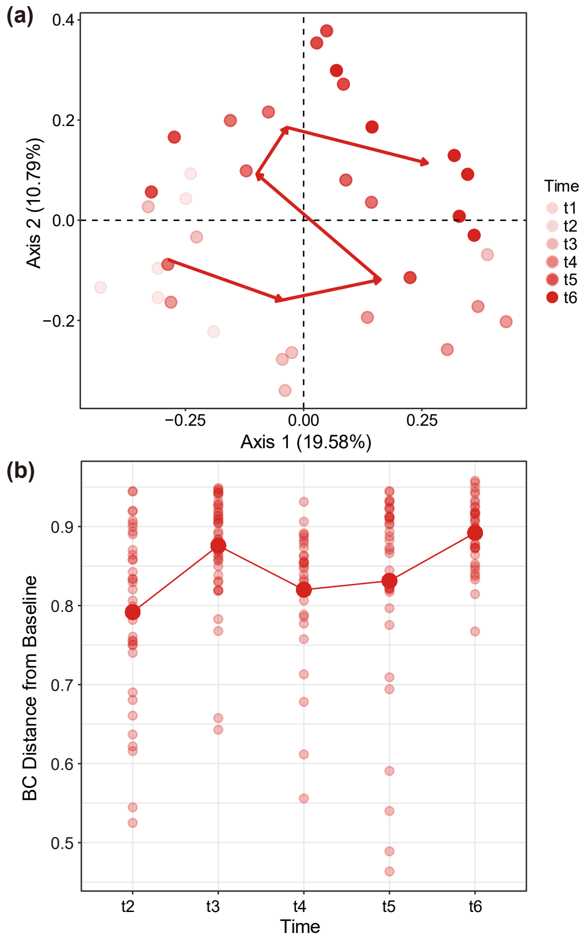


**Supplementary Fig. 11 | The changes in bacterial community structure over time.** **(a)** Cluster analysis of community similarity for samples from six time points based on Bray-Curtis distance. **(b)** Bray-Curtis distance of bacterial community from baseline (t1) at other time points (t2, t3, t4, t5, t6).

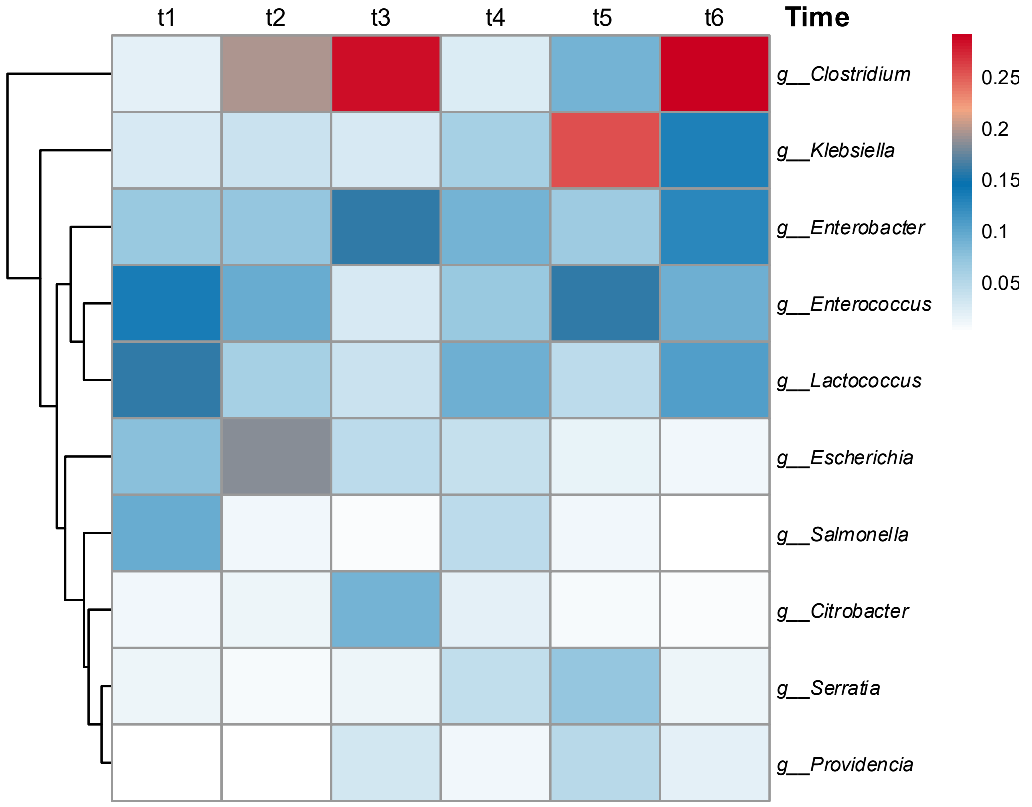


**Supplementary Fig. 12 | Bacterial genera with significant changes in abundance among six time points.** The color bars on the heatmap represent log2 fold change.

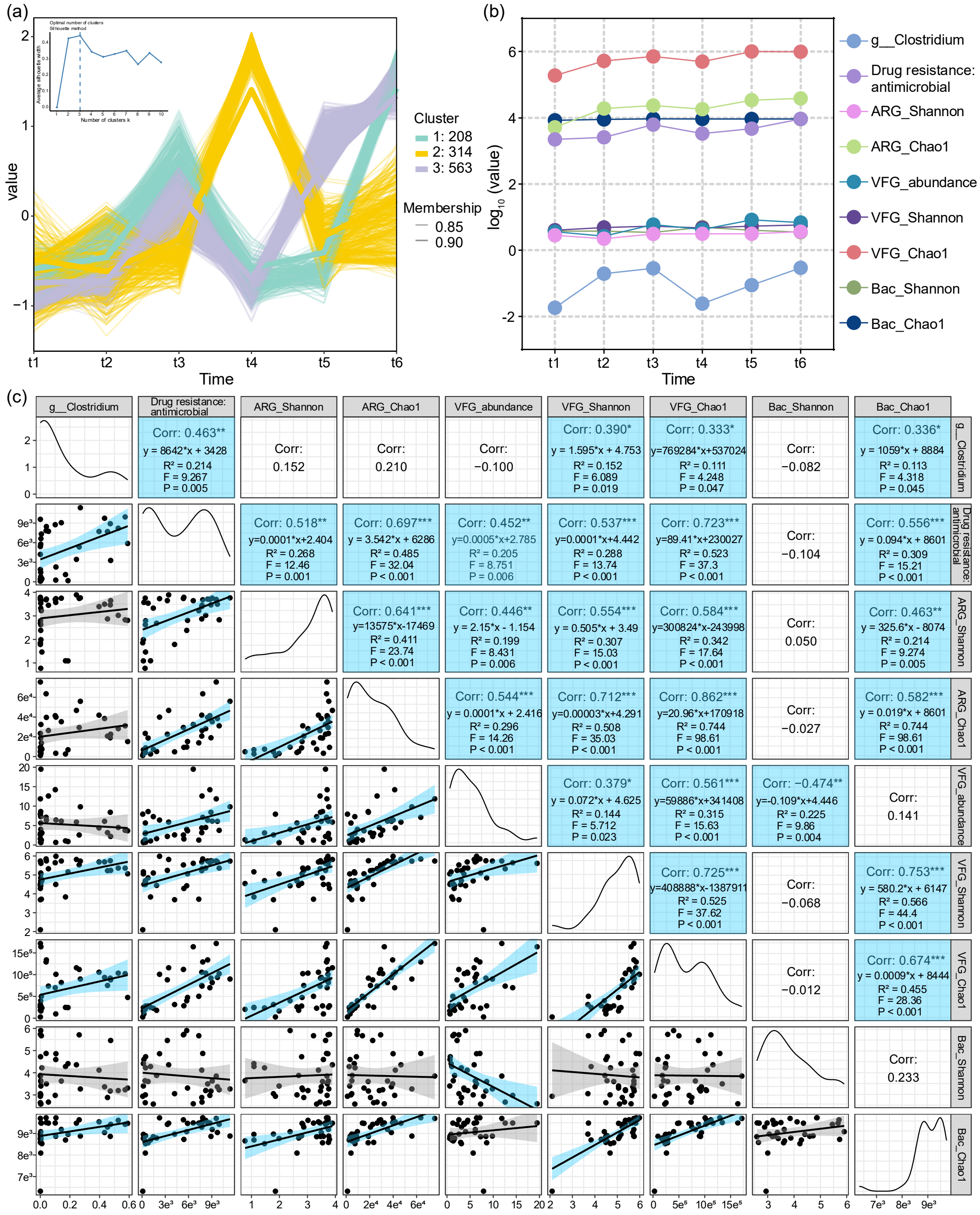


**Supplementary Fig. 13 | The relationship between abundance and diversity of bacteria, KEGG functions, ARGs, and VFGs. (a)** C-means clustering of the abundance changes of KEGG functions from t1 to t6. The subgraph represents the determination of the optimal number of clusters. **(b)** The trend of changes in the abundance and diversity of *Clostridium*, antimicrobial resistance, bacteria, ARG, and VFG from t1 to t6. **(c)** Multivariate association analysis describes the relationship between the abundance and diversity of *Clostridium*, antimicrobial resistance, bacteria, ARG, and VFG.

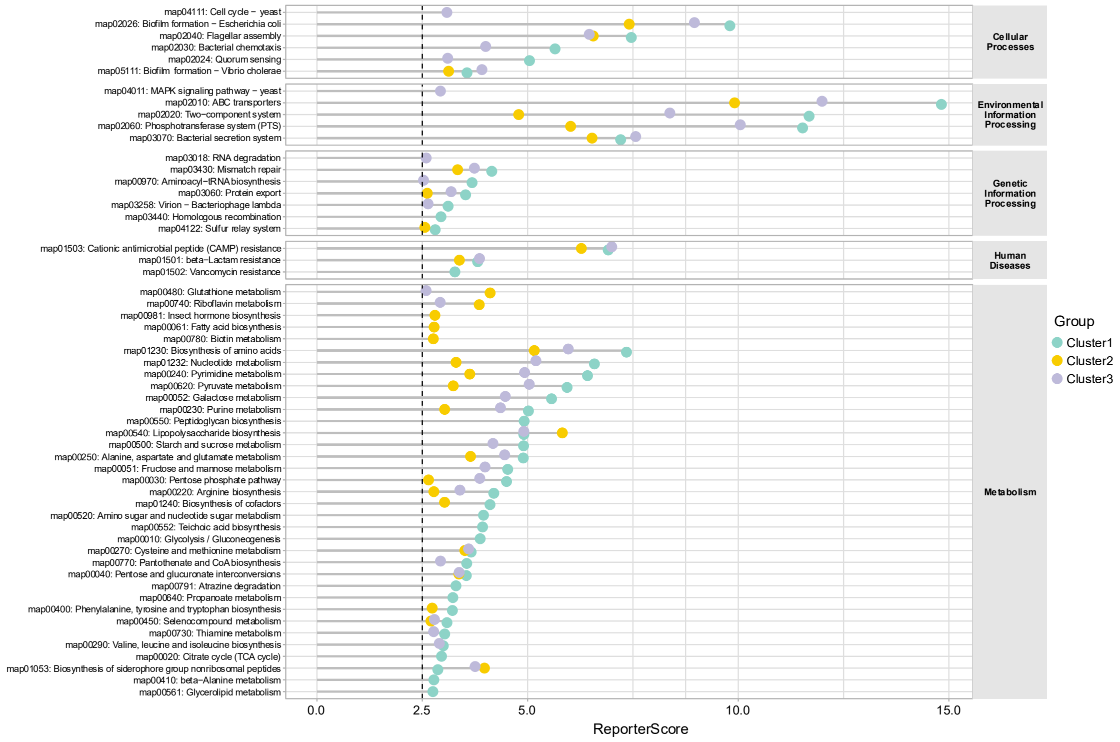


**Supplementary Fig. 14 | Enrichment analysis of KEGG functions with specific abundance variation patterns from t1 to t6.** Each cluster is determined based on previous cluster analysis.

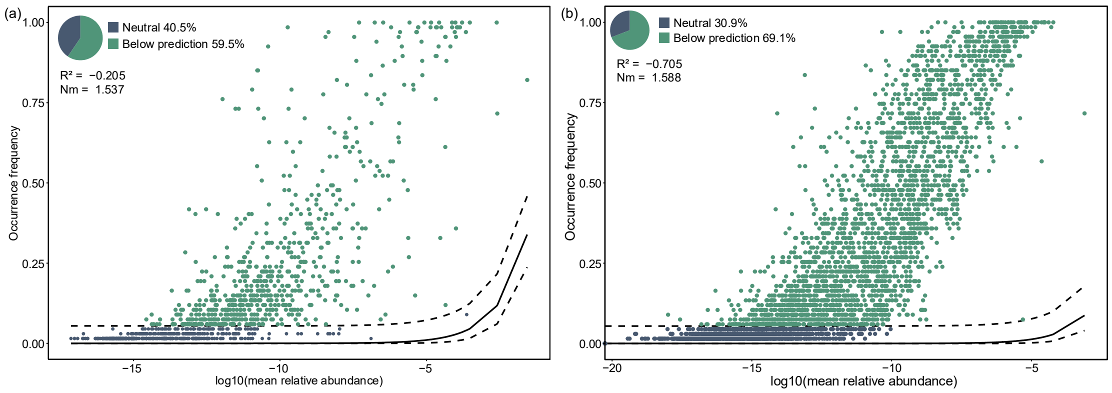


**Supplementary Fig. 15 | Community assembly mechanism of ARGs and VFGs. (a)** Fit of the neutral community model (NCM) of ARGs community assembly. **(b)** Fit of the neutral community model (NCM) of VFGs community assembly.

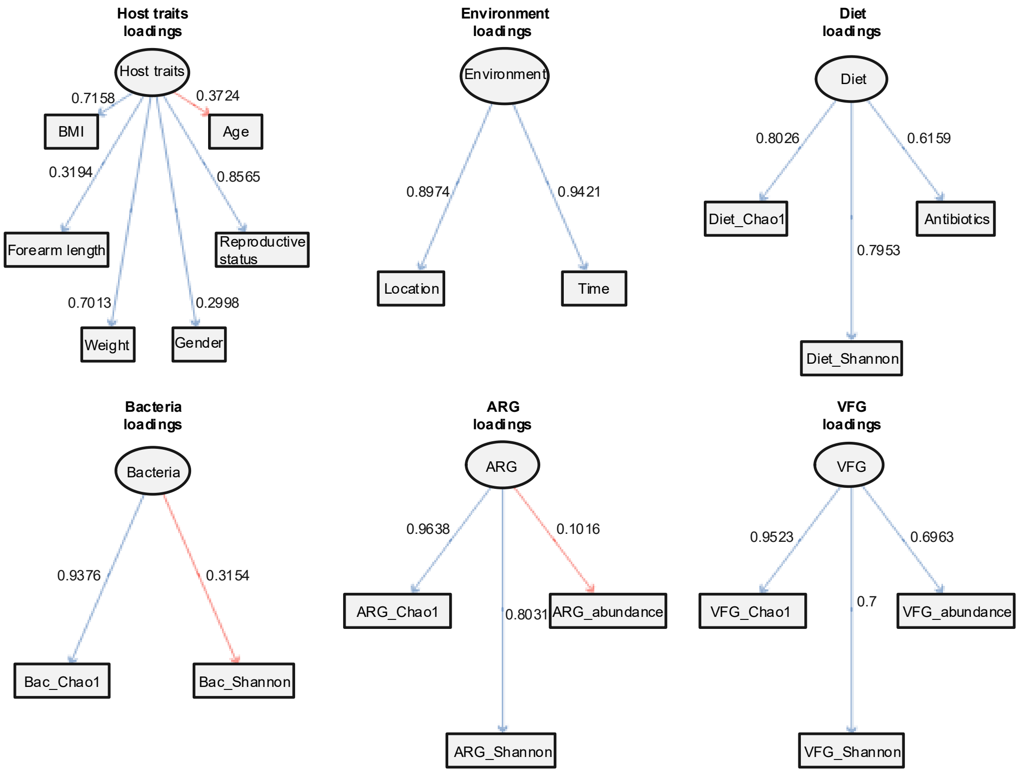


**Supplementary Fig. 16 | The external model of partial least squares path modeling (PLS-PM) in this study.** Red lines represent positive effects, and blue lines represent negative effects.

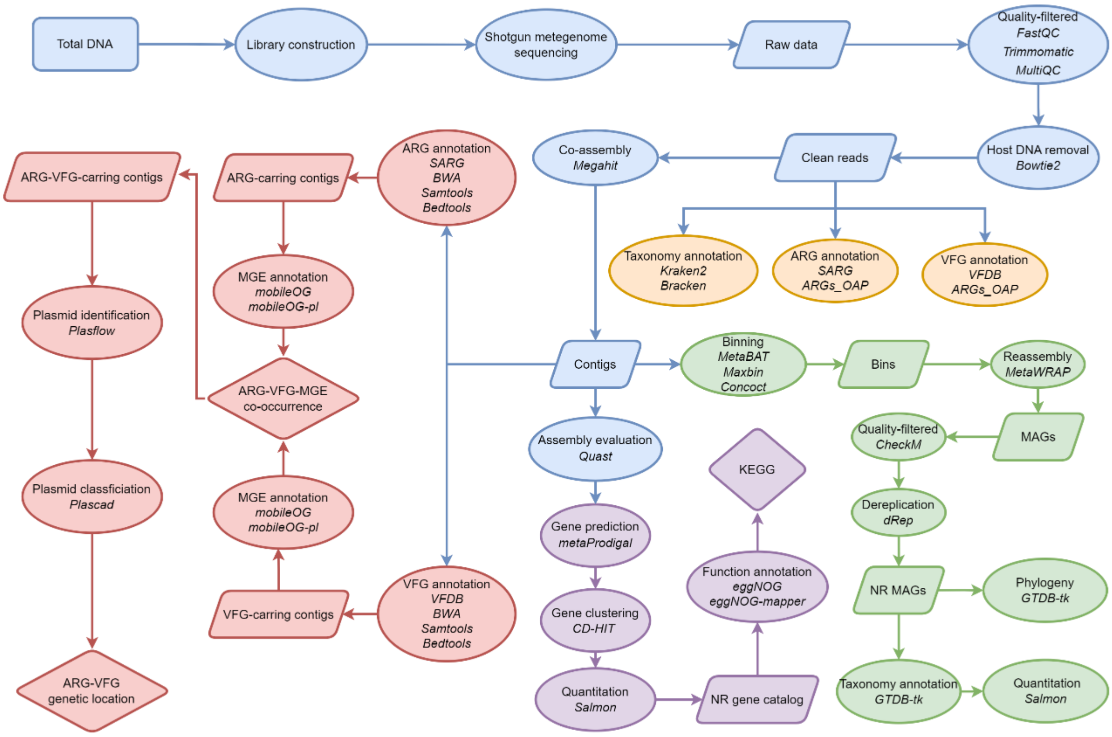


**Supplementary Fig. 17 | The bioinformatics pipeline for shotgun metagenomic data analysis in this study.**
